# Reconfiguring confined magnetic colloids with tunable fluid transport behavior

**DOI:** 10.1093/nsr/nwaa301

**Published:** 2020-12-26

**Authors:** Zhizhi Sheng, Mengchuang Zhang, Jing Liu, Paolo Malgaretti, Jianyu Li, Shuli Wang, Wei Lv, Rongrong Zhang, Yi Fan, Yunmao Zhang, Xinyu Chen, Xu Hou

**Affiliations:** State Key Laboratory of Physical Chemistry of Solid Surfaces, College of Chemistry and Chemical Engineering, Xiamen University, Xiamen 361005, China; Collaborative Innovation Center of Chemistry for Energy Materials, Xiamen University, Xiamen 361005, China; Department of Physics, Research Institute for Biomimetics and Soft Matter, Fujian Provincial Key Laboratory for Soft Functional Materials Research, Jiujiang Research Institute, College of Physical Science and Technology, Xiamen University, Xiamen 361005, China; State Key Laboratory of Physical Chemistry of Solid Surfaces, College of Chemistry and Chemical Engineering, Xiamen University, Xiamen 361005, China; Max Planck Institute for Intelligent Systems, Stuttgart 70569, Germany; IV Institute for Theoretical Physics, University of Stuttgart, Stuttgart 70049, Germany; Department of Mechanical Engineering, McGill University, Montreal H3A 0G4, Canada; Department of Biomedical Engineering, McGill University, Montreal H3A 0G4, Canada; State Key Laboratory of Physical Chemistry of Solid Surfaces, College of Chemistry and Chemical Engineering, Xiamen University, Xiamen 361005, China; Collaborative Innovation Center of Chemistry for Energy Materials, Xiamen University, Xiamen 361005, China; Department of Physics, Research Institute for Biomimetics and Soft Matter, Fujian Provincial Key Laboratory for Soft Functional Materials Research, Jiujiang Research Institute, College of Physical Science and Technology, Xiamen University, Xiamen 361005, China; State Key Laboratory of Physical Chemistry of Solid Surfaces, College of Chemistry and Chemical Engineering, Xiamen University, Xiamen 361005, China; State Key Laboratory of Physical Chemistry of Solid Surfaces, College of Chemistry and Chemical Engineering, Xiamen University, Xiamen 361005, China; State Key Laboratory of Physical Chemistry of Solid Surfaces, College of Chemistry and Chemical Engineering, Xiamen University, Xiamen 361005, China; State Key Laboratory of Physical Chemistry of Solid Surfaces, College of Chemistry and Chemical Engineering, Xiamen University, Xiamen 361005, China; State Key Laboratory of Physical Chemistry of Solid Surfaces, College of Chemistry and Chemical Engineering, Xiamen University, Xiamen 361005, China; Collaborative Innovation Center of Chemistry for Energy Materials, Xiamen University, Xiamen 361005, China; Department of Physics, Research Institute for Biomimetics and Soft Matter, Fujian Provincial Key Laboratory for Soft Functional Materials Research, Jiujiang Research Institute, College of Physical Science and Technology, Xiamen University, Xiamen 361005, China; Tan Kah KeeInnovation Laboratory, Xiamen 361102, China

**Keywords:** confined magnetic colloids, entropy, pressure threshold, fluid transport, liquid gating technology

## Abstract

Collective dynamics of confined colloids are crucial in diverse scenarios such as self-assembly and phase behavior in materials science, microrobot swarms for drug delivery and microfluidic control. Yet, fine-tuning the dynamics of colloids in microscale confined spaces is still a formidable task due to the complexity of the dynamics of colloidal suspension and to the lack of methodology to probe colloids in confinement. Here, we show that the collective dynamics of confined magnetic colloids can be finely tuned by external magnetic fields. In particular, the mechanical properties of the confined colloidal suspension can be probed in real time and this strategy can be also used to tune microscale fluid transport. Our experimental and theoretical investigations reveal that the collective configuration characterized by the colloidal entropy is controlled by the colloidal concentration, confining ratio and external field strength and direction. Indeed, our results show that mechanical properties of the colloidal suspension as well as the transport of the solvent in microfluidic devices can be controlled upon tuning the entropy of the colloidal suspension. Our approach opens new avenues for the design and application of drug delivery, microfluidic logic, dynamic fluid control, chemical reaction and beyond.

## Teaser

Collective dynamics of confined magnetic colloids are finely regulated by external magnetic fields, where mechanical properties of colloidal suspension can be probed in real-time, exhibiting tunable microscale fluid transport behavior.

## INTRODUCTION

Colloidal suspensions of microscopic particles show complex and interesting collective behaviors. In particular, the collective dynamics of colloids is fundamental and ubiquitous for materials assembly [1,2], robotic motion [3,4], microfluidic control [5,6] and in several biological scenarios [7,8]. The collective dynamics of confined colloids can be completely different from that of free colloids: for instance, confined colloids can self-organize into vortex structures [9,10], coherent motion [11] or different phase behaviors [12–14]. Through the manipulation of functional structure or swarms of confined colloids, microfluidic pumping [5,15], fluid actuation [6] or solute transport [16] could be achieved. However, due to the spontaneous and unexpected nature of colloidal suspensions, how to finely tune the collective dynamics of confined colloids remains a challenging task. Moreover, since the microscale confinement is on the same length scale as the colloidal size, it is difficult to determine how the colloids interplay with each other and the geometrical constraints. To study the colloidal collective in confinements, prior work has focused on the microscopic visualization [11,17,18] and simulation method [10,17,19–22], which lacks direct evidence to characterize the mechanical property of colloidal interaction. Can this mechanical property be probed in a direct way or expressed as feedback of force in real time? With the help of liquid gating technology, the answer could be yes. The liquid gating membrane utilizes a functional liquid stabilized by capillarity as a pressure-driven, reversible and reconfigurable gate that can seal the microscale pores in a closed state, and creates a liquid-lined pore in the open state [23–27]. Liquid gating technology has been selected as one of the 2020 Top Ten Emerging Technologies in Chemistry by IUPAC (International Union of Pure and Applied Chemistry) [28]. Although this new technology is still in its early stage, it will be enhanced greatly by the development of both solid porous materials and functional liquids with variations in their chemical and physical properties. Liquid gating technology allows certain liquids to selectively open and close pores on demand. Especially, liquid gating membranes can respond to pressure changes, which also indicates the transmembrane fluid transport capability. Therefore, utilizing the pressure-driven intrusion fluids as efficient causes, the mechanics of the confined colloids can be determined in real time.

Here, we create a system of confined colloids where their collective dynamics can be finely tuned by the magnetic field. We also establish a strategy to characterize the mechanical property of the assembled structure through the pressure-driven intrusion fluid-colloid interaction, while the fluid transport behavior can be dynamically programmed. The collective configuration of the confined colloids is statistically and thermodynamically characterized by the colloidal entropy. Meanwhile, the interplay between the confined colloids and the interplay between the colloidal suspension and geometrical constraints are simultaneously indicated by the pressure value. Both the entropy of magnetic colloids and the fluid transport are prominently affected by the geometrical constraints, packing fraction, and strength and direction of the magnetic field. Moreover, as a proof of concept, this system has been demonstrated for the application of dynamic and preprogrammed fluid transport, remote drug release, microfluidic logic, and chemical reaction, enabling sustained antifouling behavior. We anticipate that this work will enlighten the fundamental research of colloidal science, and applications ranging from fluid transport, multiphase separation and logic microfluidics, to programmable cargo transport. The findings described here would also deepen the understanding of phenomena such as the cellular collective, pollutant treatment by granular particles and stop-and-go in traffic jamming.

## RESULTS AND DISCUSSION

### Establishment of a confined magnetic colloid system

**Figure 1.**
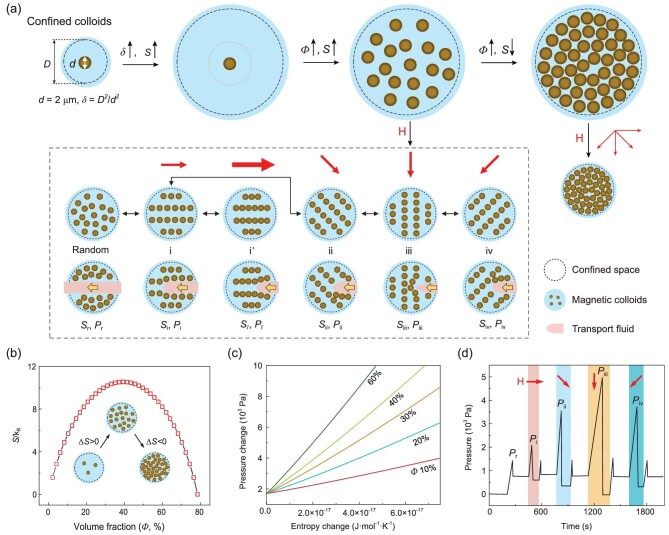
Schematics of confined colloids in different states via remote and dynamic magnetic regulation. (a) Tunable entropy of magnetic colloids in confined space and its influence on fluid transport behavior. *D* and *d* stand for the size of confined space and the diameter of the colloid, respectively. *δ* ( = *D*^2^/*d*^2^) represents the confining ratio, which is determined based on the ratio between the confinement area and the colloid area. *Φ* is the volume fraction of confined colloids. *S*_n_ and *P*_n_ (n = r, i, i’, ii, iii and iv) denote the entropy and threshold pressure for transporting the fluid. ‘r’ represents the random condition, while ‘i–iv’ stands for the condition under the magnetic field. (b) The entropy of confined colloids as a function of volume fraction. (c) Threshold pressure change of fluid transport through confined colloids concerning entropy change of the colloids. (d) Dynamic regulation of fluid transport behavior by commanding the magnetic direction. The red arrow indicates the orientation of the magnetic field.

The confined magnetic colloid system (CMCS) is formed by impregnating the magnetic suspensions (i.e. magnetorheological fluid (MRF)) in a three-dimensional (3D) foam network (Supplementary Fig. 1). MRF is a Bingham fluid, containing the microscale magnetic colloids suspended in a non-magnetic fluid and stabilized by a surfactant to avoid settlement [29]. The colloidal suspension is capillary-stabilized in the confined space. When the confined space is on the same length scale as the diameter of magnetic colloids (Supplementary Fig. 2), the freedom of the colloids is somehow limited (Fig. 1a). Entropy, relating to the organization of matter, could be brought as an apparatus of statistical mechanics and thermodynamics to understand the collective manner of colloids [30–33]. The entropy is estimated based on a lattice model [34] (Supplementary equation 1), where the volume fraction of the colloids is obtained by analyzing and accumulating the pixels of the microscopic images (Supplementary equation 2). The colloidal entropy increases as the confined space enlarges since more freedom of the colloids is provided (Fig. 1a, *δ*↑, *S*↑, where δ (= *D^2^/d^2^*) represents the confining ratio based on the confinement area and the colloid area). To quantify the interaction between the confinement and the colloids, we established a pressure-driven strategy to probe the mechanical property of the confined colloids by intruding an immiscible fluid into the CMCS and measuring the threshold of the pressure difference between the inlet and outlet of the CMCS. We find that there is a proper range to confine the colloids, i.e. *δ* = 25–625, in which the collective dynamics of confined colloids can be finely modulated and the viscosity of the colloidal suspension can be dramatically changed. In this range of confining ratio, the threshold for the intrusion fluid to permeate CMCS decreases with increasing the confinement size (Supplementary Fig. 4a–d). For a particular size of confinement, the colloidal entropy increases with the volume fraction up to a critical value (${\Phi}$↑, *S*↑), beyond which the colloidal entropy decreases with further increasing volume fraction (${\Phi}$↑, *S*↓). At the critical volume fraction, the entropy of magnetic colloids could be maneuvered in a predetermined manner. If the confined space is fully occupied with the magnetic colloids, the colloidal entropy can hardly be varied upon the external magnetic field (Fig. 1a and b). Figure 1b shows a nonlinear dependence of the entropy over the colloid volume fraction where there exists an optimized volume percentage (i.e. 40% volume fraction) for entropy regulation. The viscosity of the colloidal suspension increases with increasing the concentration of colloids (Supplementary Fig. 5a and b). The threshold pressure difference between the one with (*P*^′^) and without (*P*_0_) the magnetic field is determined as Δ*P*/*P*_0_, i.e. (*P*^′^ – *P*_0_)/*P*_0_, which increases with the weight percent of the colloids to a critical value around 70 wt.% and then decreases upon further increase of the weight percentage (Supplementary Fig. 5c). It also increases with the saturation level of infused colloidal suspension (Supplementary Fig. 4e). The pressure change is further explored as a function of the fluid flow rate, which increases as the flow rate is raised (Supplementary Fig. 5d).

### Correlation between colloidal entropy and fluid transport behavior

For the colloids with an optimized packing fraction in the confinements, we command different magnetic strengths and directions to obtain the dynamic collective structure and its mechanical property. Without the magnetic field, the colloids are randomly distributed in the confined space, with the entropy of *S*_r_. When the immiscible fluid is transported through the confined colloids, a distinct pressure threshold is required to conquer the capillary force [23–26,35], denoted as *P*_r_. In state i, under a magnetic field that is parallel to the transport flow, the confined colloids could form chain structures along the magnetic direction, during which the entropy of the colloids is reduced (*S*_i _< *S*_r_). The pressure threshold increases when the transport fluid permeates this system (*P*_i_) due to the increment of suspension viscosity. As the magnetic strength is enhanced under the same direction, the entropy is further lowered and meanwhile, the critical pressure is further increased (*S*_i’_ < *S*_i _< *S*_r_, and *P*_i’_ > *P*_i _> *P*_r_). Upon the applying of the magnetic field, the colloids aggregate to form a chain structure with minimization of Helmholtz free energy, which is comprised of magnetic energy, surface energy and energy contributed by entropy (Supplementary equation 3). The threshold pressure change of fluid transport through confined colloids and the entropy change of colloids, which are both linear with the square of magnetic strength (Supplementary equation 7, and Supplementary equation 12), are in positive linear correlation (Fig. 1c and Supplementary equation 15). The slope increases with the increasing volume fraction of colloids, providing a physical insight between the colloidal dynamics and fluid dynamics. Furthermore, we could command variant magnetic directions to manipulate the fluid transport behavior (states i–iv). By steering the magnetic direction from parallel (i), inlined (ii and iv), to perpendicular to (iii) the flow, the threshold pressure for transport fluid is gradually increased, while the entropy in a circular confined space is rarely changed (*S*_iv _= *S*_iii _= *S*_ii _= *S*_i_, *P*_iii _> *P*_ii _= *P*_iv _> *P*_i_). Once the magnetic field is off, the confined colloids reconfigure back to a random state due to the thermal energy, therefore the fluid transport behavior is also reconfigurable. Moreover, the pressure change can be dynamically tuned via the magnetic directions (Fig. 1d).

### Lattice model to illustrate the collective dynamics

**Figure 2.**
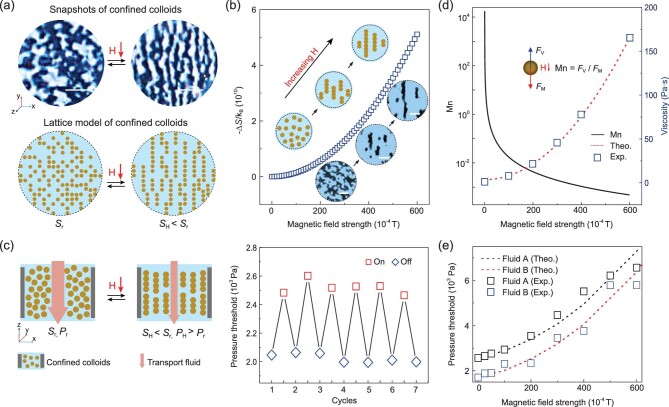
Entropy change of confined colloids in response to the magnetic field and its relevance to fluid transport behavior. (a) Snapshots of confined colloids without and with the magnetic field (H = 300 Gs), and lattice model simulation of their entropy. Scale bars: 10 μm. (b) The correlation between entropy change and magnetic field strength. Insets show the schematics and snapshots of the chain structure change. Scale bars: 10 μm. (c) Entropy change of confined colloids upon the magnetic field and the influence on fluid transport behavior, where the pressure threshold for gas could be alternately modulated by turning the magnetic field on and off. (d) The Mason number, and viscosity of magnetic suspension versus various magnetic strengths. (e) Influence of magnetic strength on the pressure threshold of gas and liquid. Fluid A represents the liquid and fluid B represents the gas.

To visualize the entropy modulation of the colloids, we confined the MRF in a transparent PDMS (Polydimethylsiloxane) pore and tuned the collective behavior of the colloids by a permanent magnet (Fig. 2a). The magnetic colloids exhibit typically soft magnetic properties that can be easily magnetized (Supplementary Fig. 3a and b). The configurations of magnetic colloids confined in a pore were observed, showing essential features of the colloids with and without the magnetic field (Fig. 2a). Meanwhile, the entropy of the colloids under the two circumstances was computed based on a lattice model simulation. In the absence of a magnetic field, the magnetic colloids are randomly dispersed in the pore with the entropy of *S*_r_. Once the magnetic field is applied, the colloids attain a magnetic dipole moment that aligns and aggregates them into chain structures. In this condition, the colloids are finally stable to acquire the minimal Helmholtz free energy, which often occurs in milliseconds [36]. The entropy is thus lowered (*S*_H_) from the lattice model (*S*_H _*< S*_r_). Based on our simulation, the increment of magnetic field strength increases the entropy change and the length of the chain (Fig. 2b). We then utilize the entropy regulation of confined colloids to manipulate the fluid transport. The MRF was infiltrated into a copper (Cu) foam forming a confined magnetic colloid system and an immiscible fluid was driven into this system where the critical pressure for transport fluid was determined. Under the magnetic field, the colloids are collected forming chains and the viscosity of the MRF is remarkably scaled up (Supplementary Fig. 1c–e). This effect increases the critical pressure (*P*_H_) and the hindrance of fluid transport (*P*_H _*> P*_r_). Moreover, the threshold pressure of transport fluid quantitatively demonstrating the force of the colloids’ interaction could be alternately modulated by changing the states of the magnetic field (Fig. 2c, right panel).

### Magnetic influences on fluid transport

To elucidate the underlying mechanism of magnetic regulating fluid transport behavior, microscopic modeling of MRF is conducted by inspecting the formation and dissociation of chain structures in the carrier fluid to predict the yield stress. The forces on the particles that govern the chain formation involve viscous force and the interparticle magnetic force. The ratio of viscous force to magnetic force is known as Mason number, Mn (Supplementary equation 8) [29]. The Mason number is inverse to the square of the magnetic field strength (Fig. 2d, black line). The inset illustrates the viscous force *F*_V_ and the magnetic force *F*_M_ on the magnetic particle under the magnetic field. The viscosity of the MRF increases due to the increment of magnetic strength, following a theoretical model (Fig. 2d, the blue square and red dashed line). The viscosity versus Mason number is plotted in Supplementary Fig. 7a.

The viscosity and shear stress versus shear rate without and with a magnetic field of 300 Gs are shown in Supplementary Fig. 3c and d. The viscosity of MRF under the magnetic field is ∼500 times higher than that without the magnetic field. The CMCS was aligned in the middle of the bar magnet with a countersunk hole, whereas the magnetic field amplitude was measured with a gauss meter and meanwhile numerically simulated (Supplementary Fig. 6a and b). The saturation time (i.e. accumulated time till permeation) and threshold pressure change *P* increase with intensifying the magnetic field (Supplementary Fig. 6c). The chain formation upon the magnetic field leads to the viscosity rising of MRF, which in turn defers the fluid transport and raises the pressure threshold. To further examine the magnetic influence on fluid transport behavior, both gas and liquid were transported through the CMCS at different magnetic strengths. The pressure thresholds of gas and liquid both increase with the magnetic field strength (Fig. 2e). We constructed a model to interpret the correlation between the yield stress of the MRF and the pressure threshold [23,37]:

(1) \begin{equation*} P = \frac{{\mu hQ}}{{kA}} + \frac{{8L}}{{\pi d}}{\tau _y}, \end{equation*}

where *μ* is the viscosity of transport fluid, *k* is the permeability of the porous medium, *h* is the thickness of porous media, *A* is the cross-sectional area of the flow, *Q* is the flow rate, *L* is the effective shear length, *d* is the effective pore size of the porous matrix and *τ_y_* is the yielding stress of the MRF (Supplementary equation 13). This analytical model is in accordance with the experimental results (Fig. 2e). Moreover, based on the function between entropy and magnetic field (Fig. 2b, Supplementary equation 7), we established the correlation between pressure threshold difference and entropy change of magnetic colloids, i.e. Δ*P* ∝ ΔS (Fig. 1c).

### Orientation control of confined colloids for fluid manipulation

**Figure 3.**
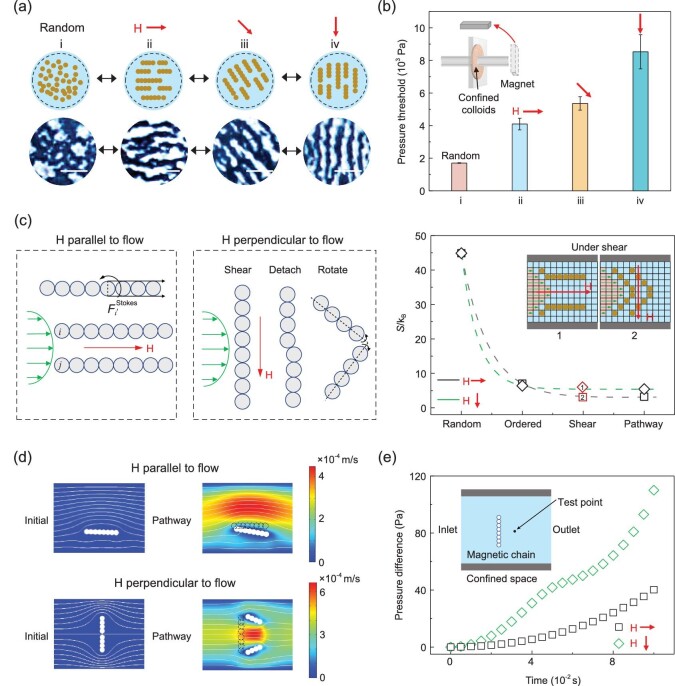
Entropy modulation via magnetic direction and the effects on fluid transport behavior. (a) Schematics and snapshots of confined colloids by tuning magnetic directions. Scale bars: 10 μm. (b) Magnetic direction dependence of gas pressure threshold. The sample is a Cu foam (diameter of 25 mm and thickness of 985.7 μm) infiltrated with MRF. Error bars indicate SD (Standard Deviation); *n* = 3 tests with the same sample. (c) Moment of inertia (left panel and middle panel) and entropy change (right panel) of confined colloids when the magnetic direction is parallel to and perpendicular to the flow. Inset shows the lattice model simulation when the confined magnetic colloids encounter the shear flow. (d) Simulation of velocity field map when the magnetic field is parallel to and perpendicular to the flow. The particles in the dashed line indicate their initial positions. (e) Comparison of pressure difference in above two conditions.

Additionally, the orientation of confined magnetic colloids could be efficiently maneuvered in a predetermined manner by varying the magnetic direction, and therefore the fluid transport behavior could also be modulated with the anisotropy of the colloids. Figure 3a shows schematics and snapshots of variant orientations of confined colloids under the same magnetic strength. In the absence of a magnetic field, the colloids are randomly dispersed. Once the magnetic field is applied, the colloids are aligned to the magnetic direction in milliseconds and the orientation can be dynamically tuned by switching magnetic directions. During the directional manipulation, the configurations can be reversely modulated (Supplementary Fig. 8 and Supplementary Movie 1). To further explore the influence of magnetic directions to the flow transport behavior, we applied three different magnetic directions to the flow direction through the CMCS (i.e. parallel to, inclined to and perpendicular to) and then transported gas into the system. The critical pressure for gas through the system increases with switching the magnetic direction from parallel to perpendicular to the flow direction, quantitatively demonstrating the force of the colloids’ interaction arisen from the magnetic anisotropy (Fig. 3b). Compared with the condition without a magnetic field, the pressure change and saturation time both increase as the magnetic direction is steered from parallel to perpendicular to the flow (Supplementary Fig. 9a and b). This could be explained by the anisotropy of the magnetoviscous effect [38], i.e. the magnetic field applied perpendicular to the flow direction (H || Q) would cause a larger viscosity than that along the flow direction (H ∥ Q).

To illustrate this, we explored the following two extreme cases (H ∥ Q and H || Q) to explain the anisotropy of the magnetoviscous effect. We simplified the whole process of fluid transport with the lattice model (Supplementary Fig. 9c and d). For both cases, the whole process is divided into four stages: (i) random, (ii) ordered, (iii) shear and (iv) pathway. In state i, the magnetic colloids are distributed randomly in the lattice. In state ii, the colloids are rearranged into chain aggregates aligning to the magnetic direction. In state iii, the MRF is invaded by a shear flow and chain-like aggregates move close to the wall side by Stokes drag force (**F**^Stokes^). In state iv, the shear flow completely pushes the magnetic colloids to the wall side, forming a stable pathway in an equilibrium state. We further analyze the difference in the invasion ability of shear flow (i.e. fluid transport behavior) in the shear state when the magnetic direction is different (Fig. 3c, left and middle panels). When the magnetic field is parallel to the flow, the fluid transport resistance is small, because the magnetic chain–chain interaction is often neglected in the equilibrium state of the chain model. Take a chain of particles that deviate slightly from the center of Poiseuille flow as an example (Supplementary Fig. 10a and b). The component **F**^Stokes^ in the direction of *z* is gradually reduced by transport flow and forms a torque rotating the chain. Therefore, the only resistance is this small rotation of the chain and its moment of inertia is approximately equal to ${I_{H\,||\,Q}} = \sum_i^n {\frac{1}{3}{m_i}} {[(i{\rm{ + }}1){a_i}]^2}$, where *m_i_* and *a_i_* are the mass and radius of the *i*^th^ particle, and *n* is the particle number in one chain (Fig. 3c, left panel), while, for the case of the perpendicular magnetic field, the **F**^Stokes^ will shear the chains directly as shown in Supplementary Fig. 10c and d. Basically, there are three steps for fluid transport in this case. Even if the **F**^Stokes^ are not large enough to break the chain, fluid transport still needs to conquer a higher resistance because of a higher moment of inertia, which can be written as ${I_{H \bot Q}} = \sum_i^n {{m_i}} {[(2i - 1){a_i}]^2}$. Here, ${I_{H \bot Q}} \gg {I_{H||Q}}$ due to *i*$\gg$ 1 in a long chain, which causes a higher hindrance in the shear flow and thus results in a big change in the magnetoviscous effect, while, if the **F**^Stokes^ are large enough to break the chain, the process is divided into four stages as mentioned before. From the force analysis, the particles integrate into chains along with the magnetic field and then are firstly sheared by the Stokes drag force of the shear flow. Secondly, chains detach at the position where the drag force is larger than the magnetic force which is dependent on the Mason number. In the third step, the detached chains rotate due to different velocities of different particles and they will reach an equilibrium state when the reformed chains move parallel to flow orientation finally (Fig. 3c, middle panel). Therefore, the critical pressure for gas flow in the case of H || Q is higher than that of H ∥ Q for more prominent magnetoviscous effect ${\eta _{H\, \bot\, Q}} \gg {\eta _{H\,||\,Q}}$.

In order to visualize the dynamics of the confined colloids when they encounter an intrusion fluid, we designed a transparent device containing a channel in a porous PTFE (Polytetrafluoroethylene) membrane mimicking the torturous pathway as in the copper foam. The magnetorheological fluid was impregnated into the channel, filling the channel and wetting the side of the channel, and airflow was pumped through the confined colloids as the intrusion fluid. Without the magnetic field and under a given applied pressure (*P*_Applied, 0_), the colloidal suspension moves along with the intrusion fluid (Supplementary Fig. 11a). Under a magnetic field perpendicular to the intrusion fluid, magnetic colloidal chains could close the flow under the same applied pressure *P*_Applied, 0_ (Supplementary Fig. 11b and b^′^). As the applied pressure increases (*P*_Applied, 1_), magnetic colloidal chains are sheared by the flow (Supplementary Fig. 11c and c^′^). With further increasing the applied pressure (*P*_Applied, 2_), magnetic colloidal chains are detached and rotated with the flow (Supplementary Fig. 11d and d^′^). When removing the magnetic field, the colloidal chains start to disappear (Supplementary Fig. 11e). Without the magnetic field, the intrusion fluid keeps flowing and forming a pathway (Supplementary Fig. 11f).

### Entropy illustration during colloidal orientation change

Among the aforementioned four states, the entropy of random, ordered and pathway states can be calculated based on the lattice model (Supplementary equations 19–23), and the entropy of shear state is somewhere between the values of the ordered and pathway states (Fig. 3c, right panel). The dimensionless entropy *S*/*k*_B_ of H ∥ Q and H || Q are equal in a random state. In the ordered state, the entropy loss of H || Q is higher than that of H ∥ Q due to the confined geometry, when the microstates of colloids in the former case are more than that of the latter. As previously mentioned in Fig. 1a, under different magnetic directions, the entropy change is the same for the colloids confined in a circular circumstance. While here, the colloids are confined in an irregular 3D network that is simplified as a cylindrical channel, the colloids have higher freedom along the transport direction than the lateral direction (Fig. 3c, inset of right panel). Higher entropy loss of ordered state shows higher shear energy consuming which means higher critical pressure. The entropy loss of H || Q is lower than that of H ∥ Q when the stable pathway is formed due to the disintegration of the chain structure. To further visualize the difference in the above two flow cases, we simulated the velocity field by COMSOL Multiphysics (Fig. 3d). It is evident that the flow velocity is higher when the magnetic field is parallel to the flow. Inversely, the pressure difference of the condition when the magnetic field is perpendicular to the flow is higher than that in the parallel case (Fig. 3e).

### Stability of CMCS

To ensure the stability of the CMCS, we examined the work of adhesion between the solid matrix (Cu foil was used here) and the colloidal suspension, the interfacial energy, and the antifouling behavior. It is proven that the magnetic suspension can adhere well to the confined network due to the optimum work of adhesion between the suspension and solid matrix (Supplementary Fig. 12). From the viewpoint of interfacial energy, it is also proven that the functional colloidal suspension is fairly stable during fluid transport both theoretically and experimentally (Supplementary Table 1, Supplementary equations 25 and 26). Additionally, the confined magnetic colloid system enables a sustainable antifouling property. For instance, the MRF-infused Cu foam could eliminate the contamination of the Rhodamine B (RB) solution, whereas the bare Cu foam is almost fully polluted by the RB solution.

### Applications of CMCS

**Figure 4.**
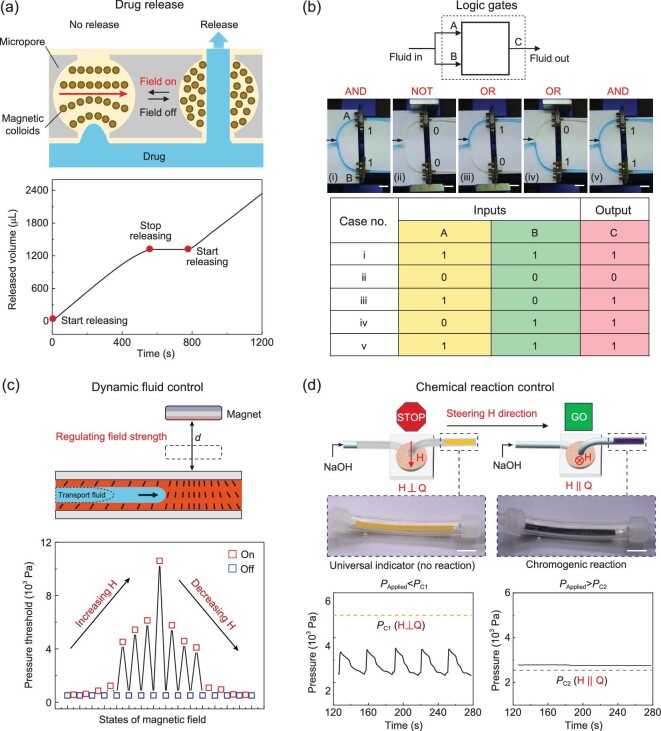
Application demonstration of CMCS. (a) Remote regulation of drug release. Under the magnetic field, the drug is impeded. Without the magnetic field, the drug is released. The released volume is shown in real time when the field is turned ‘off’, ‘on’ and ‘off’. (b) Remote modulation of microfluidic logic application. Block representation of the logic gate indicates two inputs for fluid in and one output for fluid out. The images show five logic states from AND, NOT, OR, OR to AND gate. Scalec bars: 20 mm. The truth table illustrates five corresponding states. (c) Dynamic fluid control by modulating the magnetic strength. The magnetic strength is 0, 25, 50, 100, 200, 300, 400, 500 and 600 Gs, respectively. Gs stands for Gauss (10^−4^ T). (d) Chemical reaction control by steering the magnetic direction. The scheme demonstrates that when the magnetic field is perpendicular to the flow direction, no reaction happens due to the fact that the critical pressure of NaOH is above the applied pressure (*P*_Applied _< *P*_C1_, *P*_Applied_ = 3 kPa). By steering the magnetic direction parallel to the flow that reduces the critical pressure of NaOH (*P*_Applied _> *P*_C2_, *P*_Applied_ = 3 kPa), a chromogenic reaction happens to change the color of indicator from yellow to purple. Insets show the images of the reaction chamber. Scale bars: 8 mm. The two pressure-versus-time curves correlate to the two conditions when the magnetic field is perpendicular and parallel to the flow.

Finally, we illustrate the application of CMCS by demonstrating the drug release control, logic control, dynamic fluid control and chemical reaction control. When the magnetic field is on, the confined magnetic colloids work as a physical barrier that impedes the permeance of the drug. Once the field is off, the confined colloids redistribute in disorder with a lowered apparent viscosity, providing an open pathway for the strong flux of drugs (Fig. 4a, top panel). Therefore, the released volume of the drug is modulated by controlling the ‘on’ and ‘off’ states of the magnetic field (Fig. 4a, bottom panel, Supplementary Movie 2). Moreover, if we combine two CMCSs to control the input of a microfluidic circuit, multi-logic states are achieved, including AND gate, NOT gate and OR gate (Fig. 4b). Without the magnetic field, both ports A and B permit the flow, forming an AND gate. If both port A and B are under the magnetic field, both port A and B stop the flow, achieving a NOT gate. When the magnetic field is applied to one way only, either port A or port B allows the permeability of flow, showing an OR gate. If both fields are off again, the flow will permeate through both ports again to achieve the AND gate (Supplementary Movie 3).

Furthermore, dynamic fluid control can be realized by merely tuning the magnetic strength (i.e. the distance between the magnet and the device) while keeping the fluid flow (Fig. 4c). The flux of the permeated flow reduces with finely increasing the magnetic strength, exhibiting the increment of the pressure threshold, and vice versa. Besides, by varying the magnetic direction, we can finely control a chemical reaction (Fig. 4d and Supplementary Movie 4). To demonstrate this, we design a transparent chamber containing a color indicator, which changes the color upon feeding the acidity or alkalinity of solutions. Exemplified by a chromogenic reaction, by merely steering the magnetic direction, the pressure threshold of sodium hydroxide solution (NaOH) would be decreased, and the solution would flow through the chamber to induce the chromogenic reaction, and change the color from yellow to purple.

## CONCLUSION

To sum up, we have established a system to program the collective dynamics of confined magnetic colloids, and probed the mechanical property of the colloids via intrusion fluid-colloid interaction, demonstrating tunable fluid transport behavior. Through the remote magnetic control of confined magnetic colloids, the entropy of the colloids could be modulated, and meanwhile, the pressure threshold of the transport fluid is simultaneously tuned. The entropy change of the confined magnetic colloids is closely related to the confinement, packing fraction of the colloids, magnetic strength and magnetic direction. Our models explore the synergy of thermodynamics and statistical mechanics to understand and control the collective dynamics of confined magnetic colloids. Through the intrusion liquid-colloid interaction, the colloidal interaction strength in confined space is interpreted as the pressure threshold, providing a direct strategy for characterizing the colloids’ mechanical property quantitatively. Therefore, this strategy allows us to not only qualitatively control the entropy of the confined magnetic colloids, but also quantitatively determine the mechanical property of the collective structure. Beyond the magnetic field, the reported strategy of entropy regulation of confined colloids is also applicable to other remote external stimuli, such as acoustic field, light field, electric field and so on. It is envisioned that the concept of reconfigurable magnetic colloids would find uses in smart gating valves in microfluidics, on-demand drug release, dynamic fluid transport, multiphase separation and cargo transport, and the technologies developed here would benefit areas such as swarm intelligence, the cellular collective behavior in biology, pollutant treatment via colloidal particles, and biomedical applications.

## METHODS

### Materials

Magnetorheological suspensions (MF-112) were purchased from Henan Huya Trading Co., Ltd. The density of MF-112 is 2.5 g/cm^3^. The magnetic spherical particles were carbonyl iron particles with a particle size of 2.3 ± 0.2 μm. The CMCS was established by impregnating the colloidal suspension in a copper foam. The copper foam is a cellular solid with a porous interconnected network. The copper foams were purchased from Soochow Jiashide Co., Ltd. Copper foams with an average pore size of 10 μm, 20 μm and 50 μm were used. The magnetorheological suspension was infused in the copper foam with a volume of 300 μL, 400 μL and 600 μL (400 μL was used unless otherwise stated). The magnetorheological suspension with weight percentages of 10%, 25%, 37.5%, 50%, 75%, 80% and 100% was prepared. The suspension with the weight percent of 75% was used throughout the paper unless otherwise stated. The copper foams with an average pore size of 20 μm were used for confining MF-112 suspension unless otherwise stated.

### Measurements of pressure thresholds

The aforementioned CMCS was sealed with two polymethyl methacrylate (PMMA) sheets. An inlet tube and an outlet tube were set on the two sides of the PMMA sheet. The fluid transport behavior of the confined colloidal suspensions was determined by measuring the pressure threshold during the flow of gas and liquid. Air was used as the transport gas and deionized water (resistivity 18.2 MΩ · cm) was used as the transport liquid. The pressure difference between the inlet and outlet of the CMCS was measured by wet/wet current output differential pressure transmitters (PX154–025DI and PX273–020DI) from OMEGA Engineering Inc (Stamford, CT, USA). A Harvard Apparatus PHD ULTRATM Syringe Pump was used for the flow of gas/liquid. Without specification, a flow rate of 1000 μL/min was selected for the CMCS in a constant flow rate mode.

### Reconfiguring confined colloids by the magnetic field

The magnetic field was applied to the CMCS parallel to, 45° inclined to, and perpendicular to the transport of fluid. For the dynamic response of the magnetic field, the pressure threshold was recorded without and with a magnetic field periodically for 180 s, respectively. The critical pressures of gas and liquid were measured under the magnetic field strength of 0, 25, 50, 100, 200, 300, 400, 500 and 600 Gs (1 Gs = 10^−4^ T).

### Microscopic observation of confined colloids

The microscopic view of copper foam, carbonyl iron particles and MRF in the copper foam were observed by an Olympus IX73 Microscope.

### Characterization of rheological property

Under the magnetic field, the viscosity and shear stress of MRF at various shear rates were determined by the Anton Parr MCR 302 Rheometer with a self-designed electromagnetic coil.

### Characterization of magnetic property

The magnetization versus magnetic field (B–H loop) curve and initial curve were determined by a LakeShore 7404 Vibrating Sample Magnetometer.

## Supplementary Material

nwaa301_Supplemental_Files
