# Supplementary material for: Reconfiguring confined magnetic colloids with tunable fluid transport behavior
Source: Natl Sci Rev. 2020 Dec 26;8(5):nwaa301. doi: 10.1093/nsr/nwaa301 (PMC8352900; doi:10.1093/nsr/nwaa301)
Supplement: nwaa301_Supplemental_Files [file nwaa301_supplemental_files.zip › Supplementary data.docx]

Supplementary Information

**Reconfiguring confined magnetic colloids with tunable**

**fluid transport behavior**

Zhizhi Sheng1,2†, Mengchuang Zhang3,†, Jing Liu1,†, Paolo Malgaretti5,6,Jianyu Li7,8, Shuli Wang1,2, Wei Lv3, Rongrong Zhang1, Yi Fan1, Yunmao Zhang1, Xinyu Chen1 and Xu Hou1,2,3,4,*

*Corresponding author. Email: houx@xmu.edu.cn (X. H.)

**Content：**

**1. Supplementary methods**

**2. Supplementary text**

**2.1 Mechanism and theoretical analysis of magnetic field-triggered change of colloidal entropy and fluid transport behavior**

**2.2 The anisotropy of magnetoviscous effect (MVE) by varying magnetic field orientation**

**2.3 MVE from the entropy view**

**2.4 Simulation of fluid-colloid interaction coupling with the shearing process of magnetic chains**

**2.5 Interfacial energy calculation for a stable confined magnetic colloid system**

**3. Supplementary figures**

**4. Supplementary movies (Movie 1~4)**

**1. Supplementary methods**

**1.1 Characterization of drug release**

A methylene blue solution was taken as the target drug. It is prepared by dissolving 0.0105 g methylene blue trihydrate powders (CAS # 7220-79-3) in 250 mL DI water. The solution was pumped through the confined magnetic colloid system (CMCS) at a flow rate of 1000 µL/min and the pressure threshold was measured. A constant pressure of 2 kPa was used to drive the solution, real-time pressure was recorded in the absence of and under the application of a magnetic field (300 Gs). In the absence of a magnetic field, drug solution could permeate CMCS while it is hindered by CMCS under the magnetic field. Moreover, in both cases, the flow rate through CMCS was monitored by an Everflow sensor (OB1 Mk3) under a constant driving pressure of 2 kPa.

**1.2 Characterization of microfluidic logic gate**

Two CMCSs were assembled in a Y-shaped microfluidic circuit. The methylene blue solution was pumped through each CMCS at a flow rate of 1000 µL/min and the pressure threshold was determined. Real-time pressure was measured when a 1.5 kPa constant pressure was applied to drive the solution, with both CMCSs in the absence of a magnetic field, one CMCS under a magnetic field, and both CMCSs under a magnetic field. The magnetic strength was 300 Gs.

**1.3 Chemical reaction control via CMCS**

A transparent chamber containing a strip of the pH universal indicator was connected to a CMCS. A NaOH solution with a concentration of 0.01 mol/L was pumped through CMCS at a flow rate of 1000 µL/min under the magnetic field parallel to and perpendicular to the flow, respectively. The magnetic strength was 300 Gs. During this process, the distinct pressure thresholds under these two circumstances were measured simultaneously. Given the applied constant pressure between the two thresholds, the permeability of the NaOH solution could be manipulated to control the chromogenic reaction via steering magnetic direction. When a constant pressure of 3 kPa was used to drive the NaOH solution under the magnetic field perpendicular to the flow, the NaOH solution could not penetrate CMCS. However, when the magnetic field is steered to parallel to the flow, the NaOH solution could permeate CMCS making the chromogenic reaction happen that changes the strip color from yellow to purple.

**2. Supplementary text**

**2.1 Mechanism and theoretical analysis of magnetic field-triggered change of colloidal entropy and fluid transport behavior**

Magnetic field-triggered entropy change based on the lattice model

The lattice model is used to estimate the entropy [[1](#_ENREF_1)] of the confined magnetic colloids. The entropy relates to the total number of states Ω and is defined as:

(1)

where *k* is the Boltzmann constant and equals to ~1.381×10-23 J/K. The Ω is the number of all the possibilities for the particles’ arrangements, which could be calculated by the mathematical methods of the [permutation](javascript:;) [and](javascript:;) [combination](javascript:;).

Fig. 2a shows the microstructure and lattice model of the particles with and without the magnetic field. The entropy without the magnetic field is calculated as:

Assuming that all the particles were spheres and the chains were cylinders, the average radius of particles is *d=*2.84μm, the average distance of the chains is *r*=2.336 μm. The area of the circle confined area is *A* = π(*D*/2)2. The diameter of the circle confined area is *D*=100 μm. By analyzing and accumulating the pixels of microscopic images, the volume fraction is . Then the number of particles is derived as:

(2)

And the number of total lattices is 1240. The average length of chains is assumed as 3. The number Ω is equaled to the total number of possibilities of putting 258 (774/3) particles into 413 (1240/3) lattices. Fig. 2a shows one possibility of chains arrangement and entropy with the magnetic field can be calculated as (using Stirling’s approximation)

Magnetic field-triggered entropy change based on Helmholtz free energy

Assuming that the magnetorheological fluid (MRF) is confined between two glass plates with a separation *L*, and then an external magnetic field *H*0is applied perpendicular to the plate’s surface. F. Marty Ytreberg and Susan R. McKay[[2](#_ENREF_2)] predicted the size and spacing of the aggregates for a hexagonal pattern by a function that relates to *H*0and *L.*

The size of an aggregate can be determined by a minimization of the Helmholtz free energy *F*[[2](#_ENREF_2)].

(3)

*EH* is the magnetic energy and described by:

(4)

where *η*1 and *η*2 are constants, which depend on the properties of the aggregates. *H0* is the external field strength. And *d* is the average radius of particles. *ESUR* is the surface energy which depends on the surface tension of the aggregate-plate and the aggregate-fluid interfaces, respectively.

The energy which is contributed by entropy change can be described as:

(5)

where *T* is the environmental temperature. Δ*S* is the entropy change.

To analyze the relationship between entropy change and the external field, we assume that the system of all the particles reaches the equilibrium state. Thus, *d* and *b* are constants. Without the magnetic field, the energy of all particles can be described as internal energy. By absorbing the external magnetic energy, the ordered property of all particles increases, or in the description of laws of thermodynamics, the entropy decreases. According to the law of energy conservation, we have:

(6)

Then the entropy change Δ*S* is:

(7)

where *η*1=3.01×10-5 erg/ (Oe2·cm), *η*2=7.24×10-6 erg/ (Oe2·cm), or *η*1=4.75×10-18 J/ (T2·m), *η*2 = 1.14×10-19 J/(T2·m), which are obtained by fitting our lattice model. The environmental temperature T is 300 K. The entropy change versus magnetic field strength is shown in Fig. 2b. The entropy change -Δ*S* increases as the increase of *H*0.

Magnetic field-triggered change of fluid transport behavior

The Mason number Mn is the ratio of viscous force to magnetic force acted on the particle, which describes the behavior of MR fluids at the microscopic, particle level. Mn is the governing parameter of the shear response of a particle in an MR fluid and is an essential part of research on dynamic models of chain formation. It can be written as [[3](#_ENREF_3)]:

(8)

where, , and are the relative permeabilities of free space, carrier fluid, and particles, respectively, *β* is for an isolated, linearly susceptible sphere in a uniform applied field, *H* is the field strength, is the viscosity of carrier fluid, and is the shear rate.

The viscosity of MRF is as a function of the Mason number Mn:

(9)

The high shear rate limit of apparent viscosity is denoted as *η*∞. The fitted parameter Mn* is also known as the critical Mason number and corresponds to the Mason number where the low Mn asymptote of *η/η∞* intersects *η/η∞*=1. Mn* is then defined as

(10)

where *ηpl* is the plastic viscosity, *ηc* iscarrier viscosity, *τy* is the yield stress,is the reference stress [[3](#_ENREF_3)]. According to our experimental data, the fitted results are *=*3.6023 Pa·s, Mn*=0.078. The apparent viscosity vs. Mason number graph is shown in Supplementary Fig. 5. In this system,is fitted as 173.3 G2. The theoretical and experimental apparent viscosity *η* versus magnetic field strength *H* graph is shown in Fig. 2d.

With infiltrated MRF, the system is tunable by the applied magnetic field. Considering the Bingham behavior of MRF, the Darcy relation is modified as follows:

(11)

where *μ* is viscosity, *k* is the permeability of the medium, *Q* is the flow rate, *h* is the length of pressure drop and *A* is the cross-sectional area to flow. The yielding stress *τy* depends on the magnetic strength, which can be measured separately with rheometers. *L* is the effective shear length. The pressure change due to the magnetic field is as follows [[4](#_ENREF_4)]:

(12)

Yielding stress is the key factor that differs from the critical pressure. Generally, MRF is considered approximately as a type of Bingham non-Newtonian fluid, of which its yielding stress can be estimated by the micro chain-rod model [[5](#_ENREF_5)].

(13)

where *a* is the radius of magnetic particle, *δ* is the distance between each particle, *χ* is [magnetic](javascript:;) [susceptibility](javascript:;), *φ* is the fraction of porosity, *H* is applied magnetic field.

The yielding stress is expressed as a [quadratic polynomial](javascript:;) of magnetic induction *B* due to the approximate formula *B*=*μH*, which is also replaced by the empirical formula

(14)

where *C* is a constant fitting number. Supplementary Fig. 5b shows the relationship between yielding stress *τ* and magnetic induction *B* in gas transport according to equation S14. We further compared the experimental results of critical pressure versus magnetic induction B to the results obtained by formula (S11), shown in Fig. 2e.

According to equation S7, the entropy could influence the yielding stress by controlling the applied magnetic field. We can, therefore, obtain the relationship between critical pressure and entropy change by combining with equation S13,

(15)

It shows that the critical pressure is in linear relation to entropy change (Fig. 1c). We also conducted the calculation for the pressure change vs entropy change for colloids at different volume fractions. It indicates that a higher volume fraction of magnetic colloids could lead to a higher critical pressure at the same entropy change.

**2.2** **The anisotropy of magnetoviscous effect (MVE) by varying magnetic field orientation**

Anisotropy of magnetoviscous effect (MVE) is affected by the orientations of the external magnetic field. From our experiment, it shows that the magnetic field applied along the gradient of the flow direction (perpendicular to the flow) causes a larger viscosity than that along the flow direction [[6](#_ENREF_6), [7](#_ENREF_7)]. In our system, transport fluid flows through the porous media and overcomes the shear resistance by MRF to form a stable pathway, which is a typical two-phase flow shear model. The flow of MRF in micropores is [in](javascript:;) [accordance](javascript:;) [with](javascript:;) [the](javascript:;) [characteristic](javascript:;) of Poiseuille flow. We analyzed the following two extreme cases to explain the anisotropy of MVE: the magnetic field H parallel to and perpendicular to flow Q.

Magnetic field H parallel to flow Q (H||Q)

For the Poiseuille flow when the magnetic field is parallel to the flow, all particles have the same velocities which the ‘detach’ and ‘rotate’ process hardly occurs (Fig. 3c). The chains will move to the wall of micro-holes by small perturbations along with the shear flow to generate a pathway, shown in Supplementary Fig. 9c. Because the chain-chain magnetic interaction is often neglected in the [equilibrium](javascript:;) [state](javascript:;) of chains model [[8](#_ENREF_8)], assuming the chains as rod model (Supplementary Fig. 10a,b), the only resistance is the small rotation of chains and its moment of inertia is approximately equal to:

(16)

Magnetic field H perpendicular to flow Q (H⊥Q)

The system is subjected to the magnetic field and shear flow. For the Poiseuille flow when the magnetic field is perpendicular to the flow, the velocity of particles in one chain is different because the same velocity exists in the position with the same radius. Assume a chain as a rod model with 8 particles sheared by Poiseuille flow, different velocities at different radii are shown in supplementary Fig. 10d. The process can be divided into 3 steps. Firstly, the particles integrate into chains along with the magnetic field. Then, the chains are sheared by the Stokes drag force. Secondly, chains detach at the position that the drag force is larger than the magnetic force which is dependent on the Mason number. In the third step, the detached chains rotate because of the different velocities of different particles and they will reach an [equilibrium](javascript:;) [state](javascript:;) in which the reformed chains finally move parallel to flow orientation. The micro chains are disintegrated by shear flow and recombined by the magnetic field repeatedly during the whole process, shown in Fig. 3c. In the step of detaching, detaching needs a minimum drag force equal to the Coulomb force between the two parts of detached chains,

(17)

where *n* is the particle number of one chain-like aggregate, *r* is the distance between adjacent two particles, *θ* represents the angle between the magnetic field and the vertical direction, Magnetic moment, *a* is the mean radius of particles, and is Coulomb constant. Besides, in the step of rotation, the Stokes drag force still needs to overcome the larger resistance of rotation due to the large moment of inertia. Based on the assumption of the rod model (Supplementary Fig. 10c,d), the moment of inertia can be expressed as:

(18)

where *ai* is the averaged radius of particle and *n* is the number of particles in one rotating chain. Here, due to *i* >> 1 in a long chain, which causes a lower hindrance in the shear flow and thus a small change in the viscosity. Therefore, the critical pressure of the case of H⊥Q is larger than that of H||Q for the apparent magnetoviscous effect .

**2.3 MVE from the entropy view**

Here, we gave the explanation of MVE from the entropy view. Assume a 2D model with 16 iron particles in a channel with 10×12 lattices and two extreme cases of 0º and 90º between H and Q.

Magnetic field H parallel to flow Q (H||Q)

We divided the shear process into 4 states and analyzed them respectively, as shown in Supplementary Fig. 9c,d. In state i, the particles distribute freely in the lattice model without any external field, which is called the “random state”. The dimensionless entropy of state i can be computed by combination law as:

(19)

In state ii, iron particles are rearranged into chains aggregates by the external magnetic field, which is called the “ordered state”. The dimensionless entropy of state ii is similarly computed as:

(20)

The entropy reduces largely due to the increase in the order of degree. In state iii, the shear flow in the same orientation of H starts to shear the [magnetorheological](javascript:;) [fluid](javascript:;) and chain-like aggregates moved close to the wall side by Stokes drag force, which is called “shear state”. The shear state is a [dynamic](javascript:;) state that entropy computation is impossible. However, we can estimate that the entropy of MRF will be continuously decreasing because external energy is flowing into the system.

Finally, the shear flow pushes the MRF out of pores and form a stable pathway as an [equilibrium](javascript:;) [state](javascript:;), which is called the “pathway state”. The entropy of state iv is decreased by the pathway narrowing and colloids ordered by external field and can be computed by

(21)

Magnetic field H perpendicular to flow Q (H⊥Q)

Supplementary Fig. 9d shows the states of H⊥Q in the lattice model similar to the case of H||Q. In the random state, no difference was found compared to the case of H||Q and the dimensionless entropy was equal to 44.88.

In the ordered state, lattices of pore geometry in the radial direction is less than that in the flow direction. Therefore, the entropy in state ii of H⊥Q is lower than that of H||Q and can be calculated as

(22)

In the shear state, the chain-like aggregates resist the shear flow to form the pathways, which are continuously disintegrating and recombining by the effect of shear drag force and magnetic force. The uncertainty is, therefore, increasing compared to the case of H||Q and entropy will be estimated higher than that of H||Q.

In the pathway state, the disintegrated chains move to the wall side. The entropy is therefore equal to

(23)

Then we compared the entropy change of these two cases as shown in Fig. 3c (right panel) and conclusions could be found as follows:

1) Entropy is equal in the random state for both cases;

2) Entropy loss Δ*S*H⊥Q is larger than Δ*S*H||Q with the magnetic field due to pore geometry;

3) Entropy loss Δ*S*H⊥Q is smaller than Δ*S*H||Q when the stable pathway formed due to disintegration of chain microstructure;

4) Higher entropy loss of State ii shows higher shear energy consuming which means higher critical pressure.

**2.4 Simulation of fluid-colloid interaction coupling with the shearing process of magnetic chains**

In the MRF, the micro-chains of magnetic colloids under the interaction of magnetic dipole force could be sheared, moved, and rotated when [two-phase](javascript:;) [flow](javascript:;) shear occurs. And different shear orientations influence the shear force. Here, we use COMSOL Multiphysics to simulate this [fluid-colloid](javascript:;) [interaction](javascript:;) in a capillary channel for magnetic influenced chains in different shear orientations. Free channel laminar flow and elastic mechanics models are used to simulate shear flow and deformation of magnetic chains. A 2D model is established as shown in the inset of Fig. 3e (inset).

In this model, the flow channel is 20 μm high and 80 μm long. The vertical magnetic chain structure is set as a solid domain with 8 particles of 2 μm radius and sits 25 μm away from the channel’s left boundary and 6 μm from the bottom boundary. The material of particles is [carbonyl](javascript:;) [iron](javascript:;) and the displacement of the center particle is set *x*=0 for the viscosity of MRF and the capillary force from micropores. The field force is applied for the whole model to simulate the magnetic force, where *m* is the magnetic moment, *k* is Coulomb constant, *r* is the radius of the particle, *n* is the number of particles in one chain, *α* is the angle between flow and magnetic field, and **e** is the direction vector with length equals to 1. The left boundary is defined as the inlet of the fully developed laminar shear flow (here is silicon oil with a density of 0.963 g/ml and [dynamic](javascript:;) [viscosity](javascript:;) of 0.05 Pa·s) with a velocity, where. The right boundary is the outlet. The interaction is governed by the incompressible Navier-Stokes equations [[9](#_ENREF_9)],

(24)

**I** denotes the unit diagonal matrix and **F** is the volume force affecting the fluid. Assume that no gravitation or other volume forces affect the fluid so that **F** = **F**m. The coordinate system velocity is **u***m* = (*um*, v*m*). Meanwhile, a freely moving deformed mesh, which constitutes the fluid domain, is also applied in this paper. The model of chains parallel to the flow direction is also established in this way. The velocity and pressure analysis are shown in Fig. 3d,e.

**2.5 Interfacial energy calculation for a stable confined magnetic colloid system**

To ensure a stable confined magnetic colloid system, we should consider the total interfacial energies of the following configurations: a) the porous matrix is infused with the magnetorheological fluid and the transport fluid is floating on top of it (*E*1), b) the porous matrix is infused with the magnetorheological fluid (*E*2), c) the porous matrix is infused with the transport fluid (*E*3). To ensure the functional colloidal suspension has a higher affinity to the porous Cu foam than the transport liquid, one should satisfy and . andare determined by [[10](#_ENREF_10)]:

(25)

(26)

where, *R* is the roughness factor of the copper foam, expressed as the ratio between the actual and projected surface areas. , , and represent the surface tension of transport liquid, magnetorheological fluid, and the interfacial surface tension between transport liquid and magnetorheological fluid. and are the equilibrium contact angles of transport liquid and magnetorheological fluid on a flat solid copper foil surface, respectively.

**References**

1. Richard A. Vaia and Giannelis EP. Lattice model of polymer melt intercalation in organically-modified layered silicates. *Macromolecules* 1997; **30**: 7990-9.

2. Ytreberg FM and Mckay SR. Calculated properties of field-induced aggregates in ferrofluids. *Phys Rev E: Stat Phys, Plasmas, Fluids, Relat Interdiscip Top* 2000; **61**: 4107.

3. Sherman SG, Becnel AC and Wereley NM. Relating Mason number to Bingham number in magnetorheological fluids. *J Magn Magn Mater* 2015; **380**: 98-104.

4. Kuzhir P, Bossis G and Bashtovoi V *et al.* Flow of magnetorheological fluid through porous media. *Eur J Mech B/Fluids* 2003; **22**: 331-43.

5. Bossis G, Lacis S and Meunier, A *et al.* Magnetorheological fluids. *J Magn Magn Mater* 2002; **252**: 224-8.

6. Linke JM and Odenbach S. Anisotropy of the magnetoviscous effect in a cobalt ferrofluid with strong interparticle interaction. *J Magn Magn Mater* 2015; **396**: 85-90.

7. Linke JM and Odenbach S. Anisotropy of the magnetoviscous effect in a ferrofluid with weakly interacting magnetite nanoparticles. *J Phys: Condens Matter* 2015; **27**: 176001.

8. Kuzhir P, Bossis G and Bashtovoi V. Effect of the orientation of the magnetic field on the flow of a magnetorheological fluid. I. Plane channel. *J Rheol* 2003; **47**: 1373-84.

9. Albarbar A and Batunlu C. *Guide to modeling and simulation* 2018.

10. Wong T-S, Kang SH and Tang SKY*, et al.* Bioinspired self-repairing slippery surfaces with pressure-stable omniphobicity. *Nature* 2011; **477**: 443.

**3. Supplementary figures**


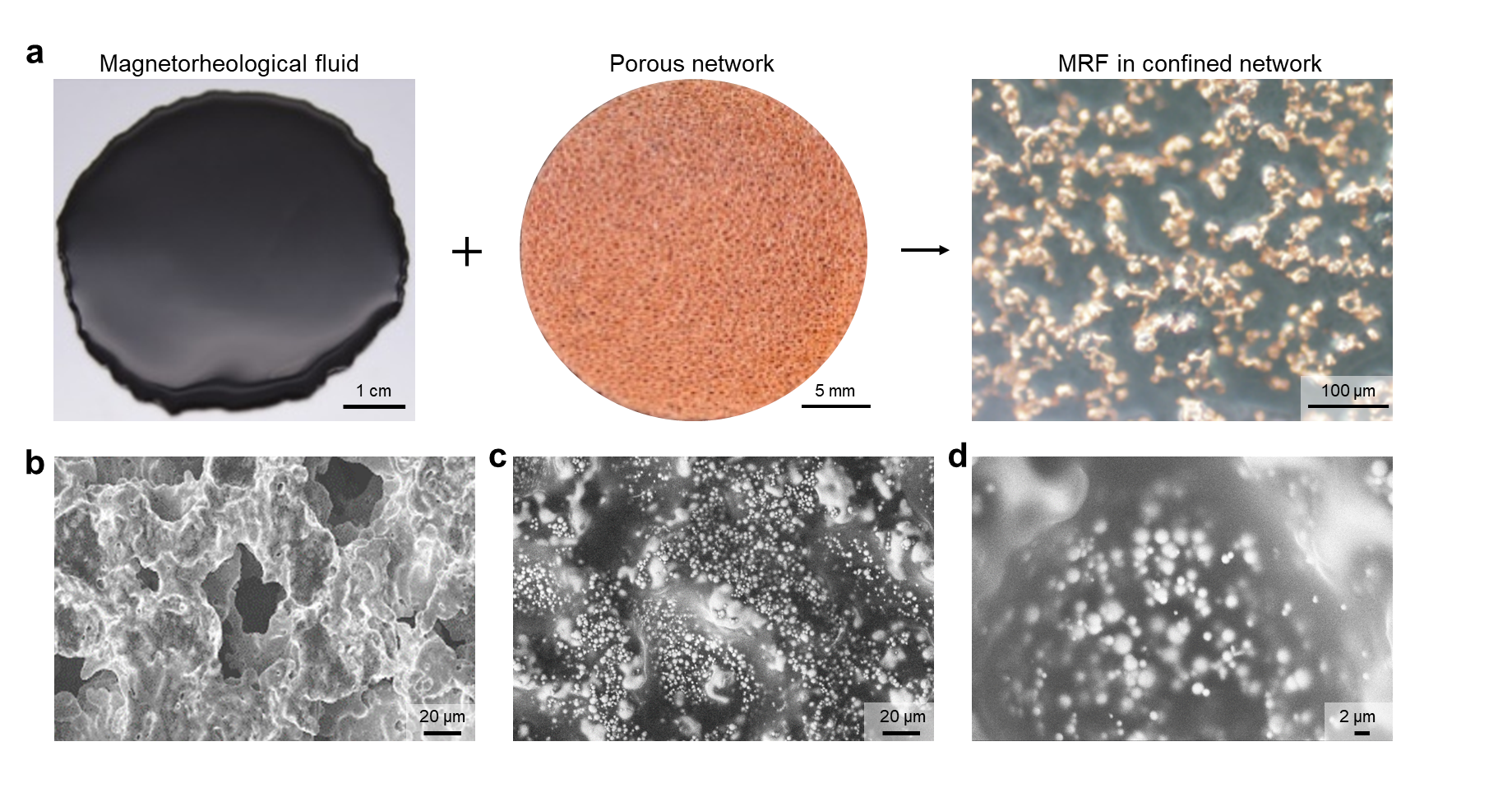


**Supplementary Figure 1. The establishment of a confined magnetic colloid system.** **a**, The confined magnetic colloid system is formed by impregnating the magnetorheological fluid in a porous network (copper foam). **b**, An SEM image of the copper foam. **c**, **d**, SEM images of magnetic colloids confined in the copper porous network.


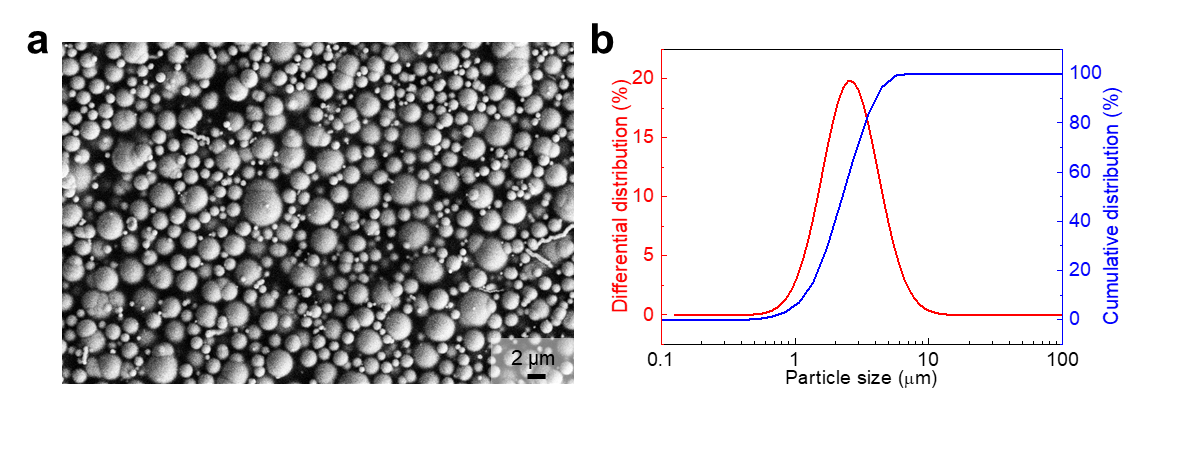


**Supplementary Figure 2. The morphology and size distribution of magnetic colloids.** **a**, An SEM image of carbonyl iron particles. Scale bar: 2 μm. **b**, The differential distribution and cumulative of carbonyl iron particles.

**
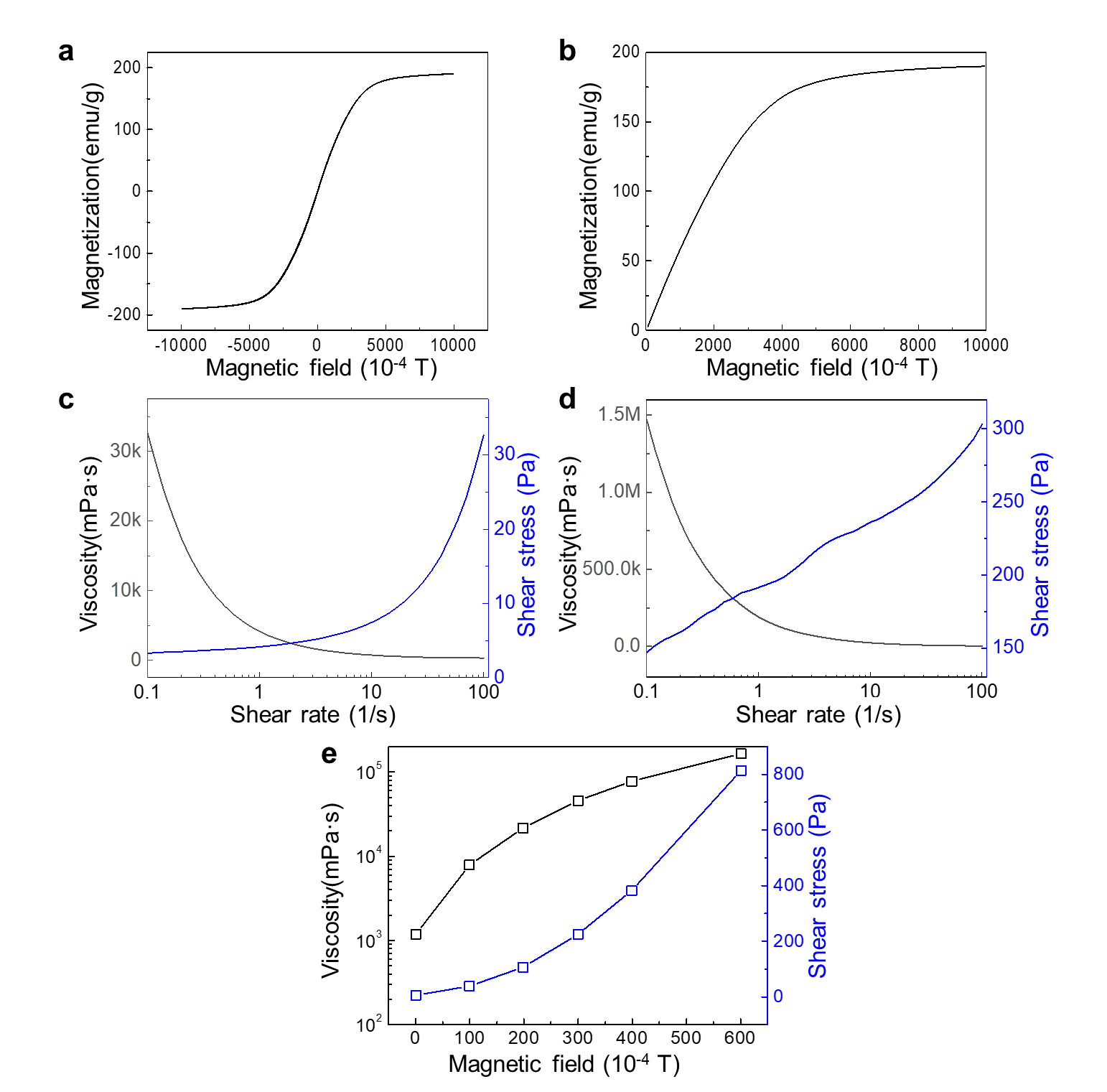
**

**Supplementary Figure 3. Magnetic property and rheological property of the magnetic suspensions. a**, The B-H curve of carbonyl iron particles. **b**, The initial magnetization curve of carbonyl iron particles. **c**, Viscosity and shear stress of MRF without the magnetic field. **d**, Viscosity and shear stress of MRF under the magnetic field of 300 Gs. **e**, Viscosity and shear stress as a function of the magnetic field.


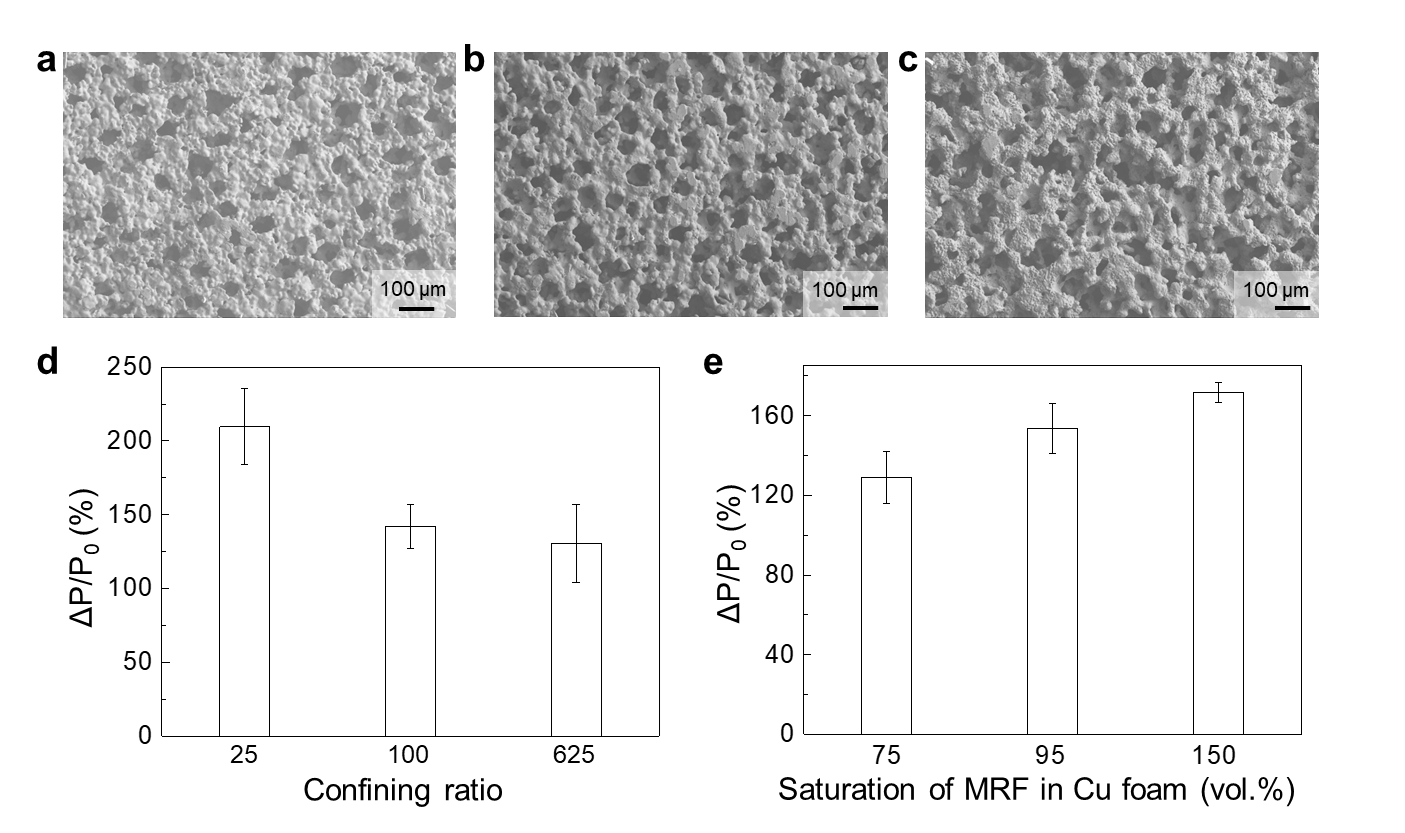


**Supplementary Figure 4. The influence of pore size on the pressure threshold of the gas. a-c,** SEM images of Cu foam in the size of 10 μm (a), 20 μm (b), and 50 μm (c). **d**, Confinement dependence of pressure change between the system with and without the magnetic field. The confining ratio is obtained from the above three different pore sizes. Error bars indicate STD; N=3 tests with the same sample. **e**, The influence of saturation of the magnetic suspension in Cu foam on the pressure change.The pore size of the Cu foam is 20 μm. Error bars indicate STD; N=3 tests with the same sample.


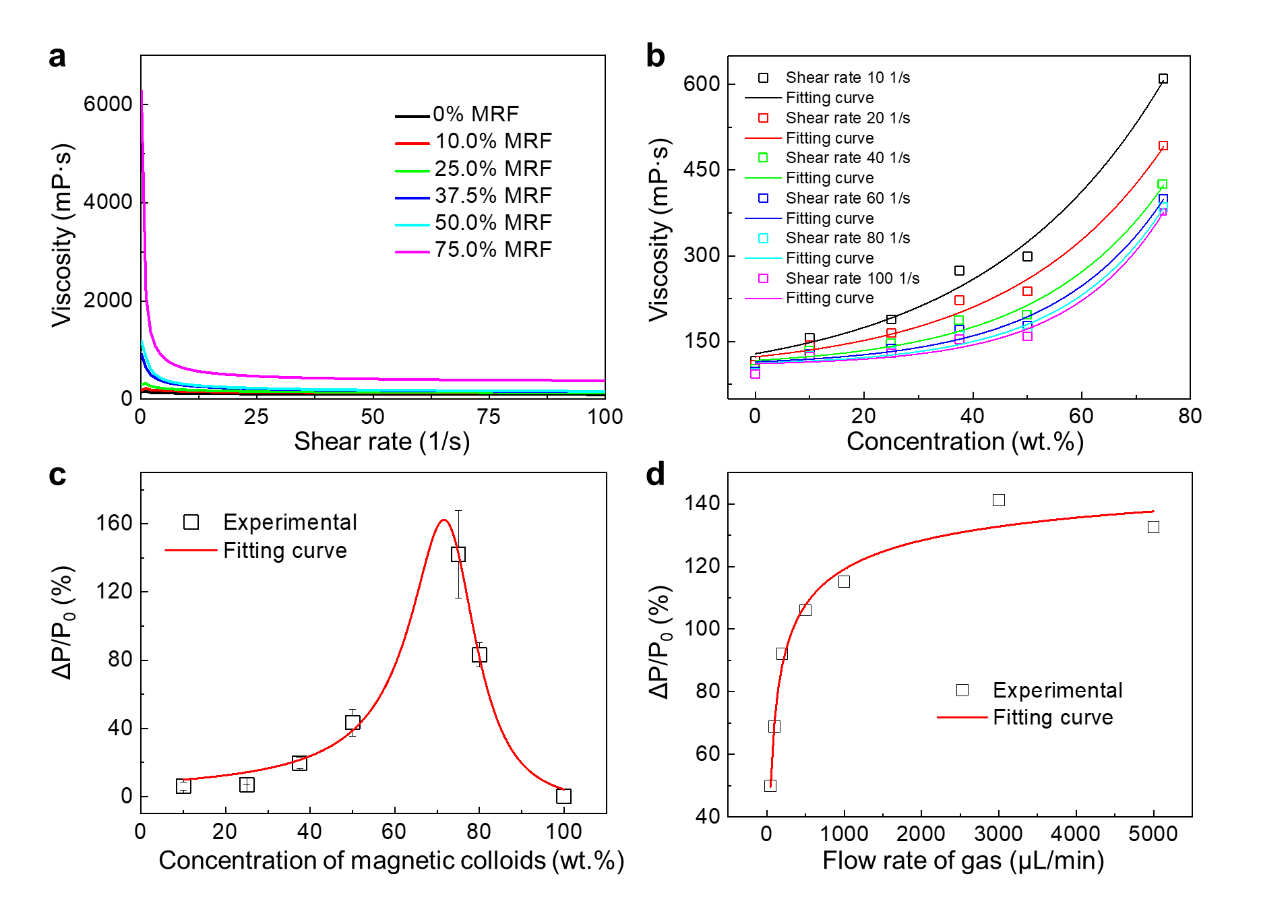


**Supplementary Figure 5. The influence of concentration of magnetic suspension, and flow rate of the transport gas on the pressure threshold change between the system with and without the magnetic field. a**,Viscosity of MRF versus the shear rate at different concentrations. **b**, Viscosity of MRF versus concentration in a series of shear rates. **c**, The influence of concentration of magnetic colloids on the pressure change.The black square represents the experimental data while the red curve is the fitting line. **d**, The dependence of gas flow rate on the pressure change**.** The black square represents the experimental data while the red curve is the fitting line.


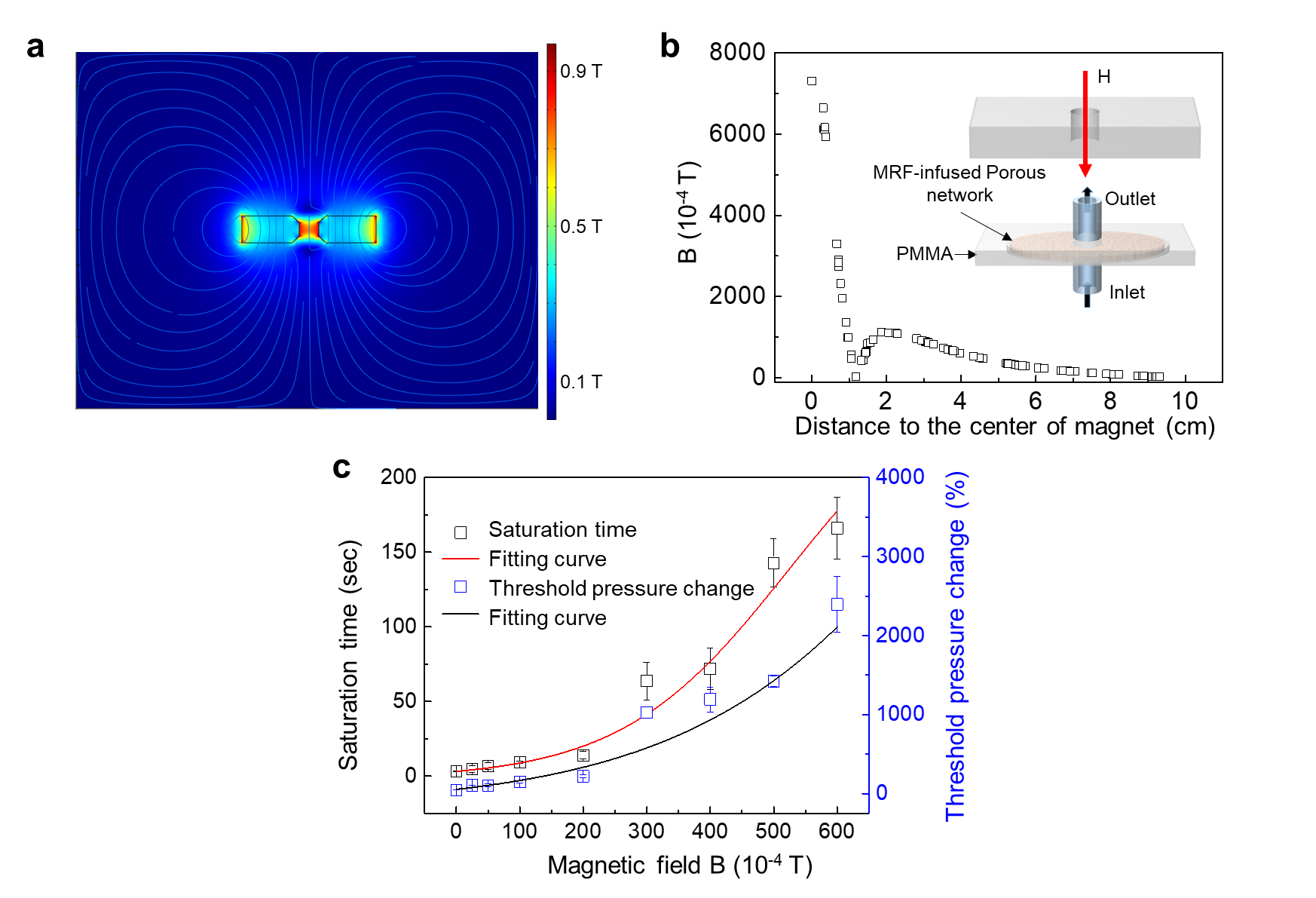


**Supplementary Figure 6. The pressure saturation time and threshold pressure change in response to the magnetic field.** **a**, The magnetic field distribution of a permanent magnet. The color scale is the magnetic field strength. **b**, Distance dependence of the magnetic field along the central line of the magnet. **c**, The influence of magnetic field on the pressure saturation time and threshold pressure change.A Cu foam with a diameter of 25 mm and a thickness of 985.7 µm is infiltrated with MRF. The pore size of the Cu foam is 20 µm. The effective area for gas transport is 28.26 mm2.


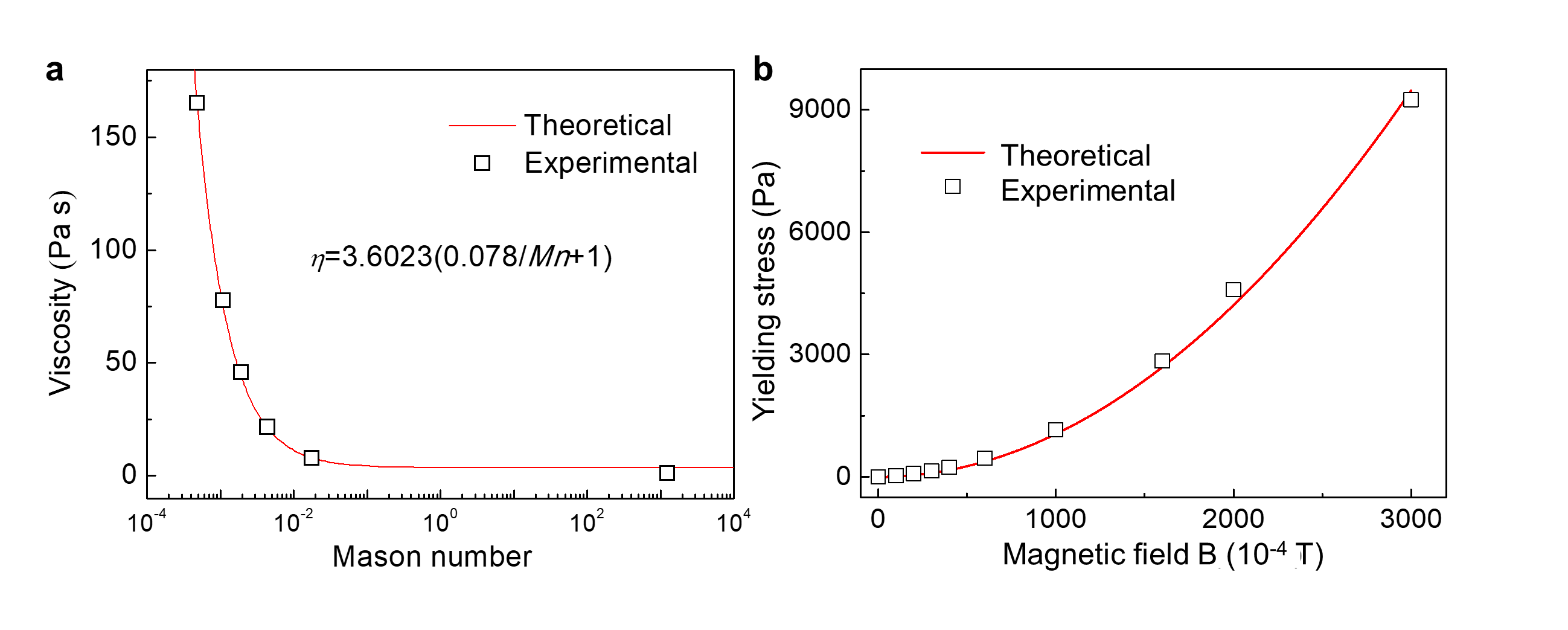


**Supplementary Figure 7. The relation between the viscosity of MRF and Mason number, and the yielding stress versus magnetic field strength. a**, The viscosity correlates to Mason number. **b**, The yielding stress as a function of magnetic field strength. The square symbol is the experimental data while the red line is the theoretical data.


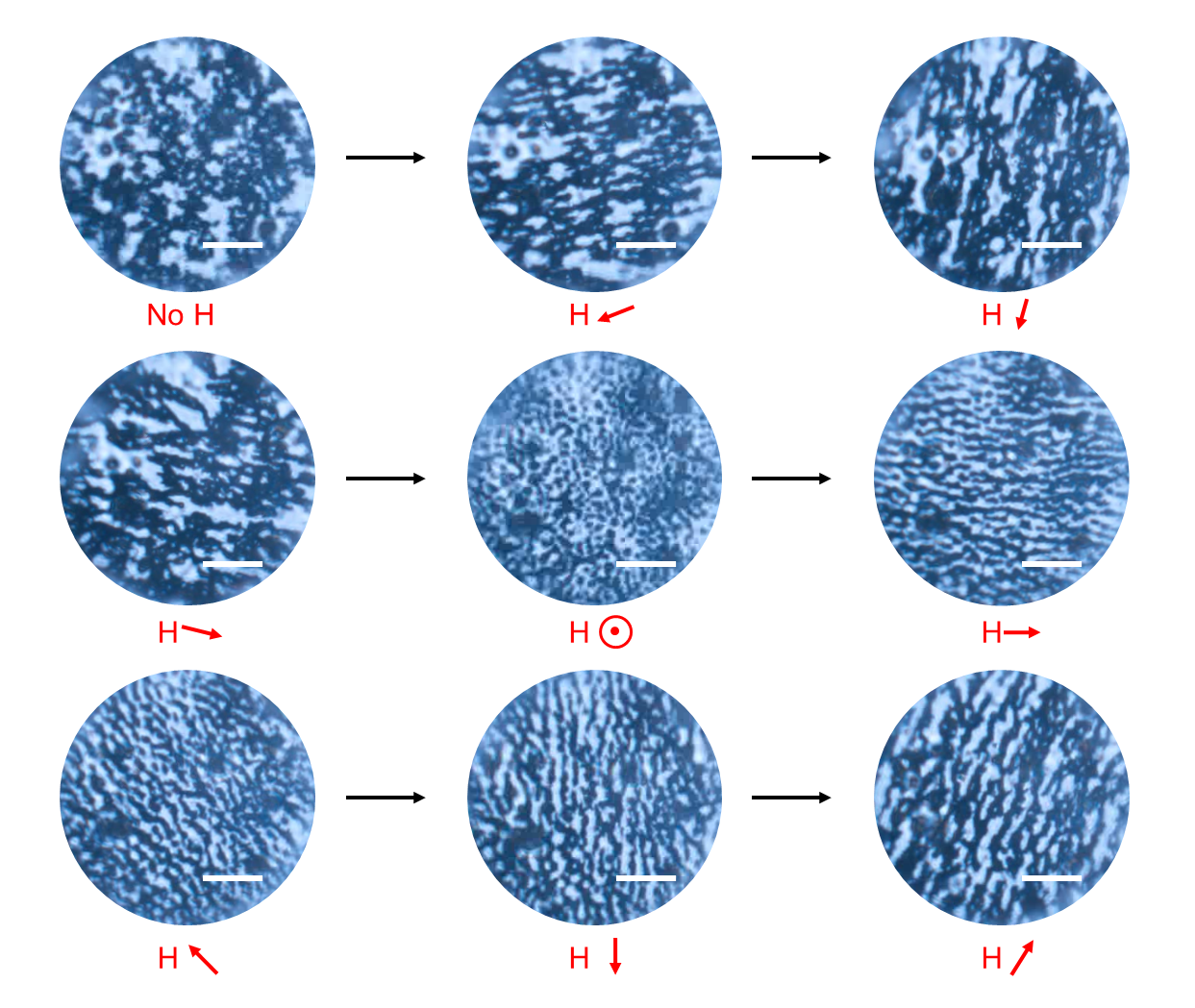


**Supplementary Figure 8. The magnetic field-induced configurations of MRF confined in a 100 μm-PDMS pore.** Scale bar is 25 μm.


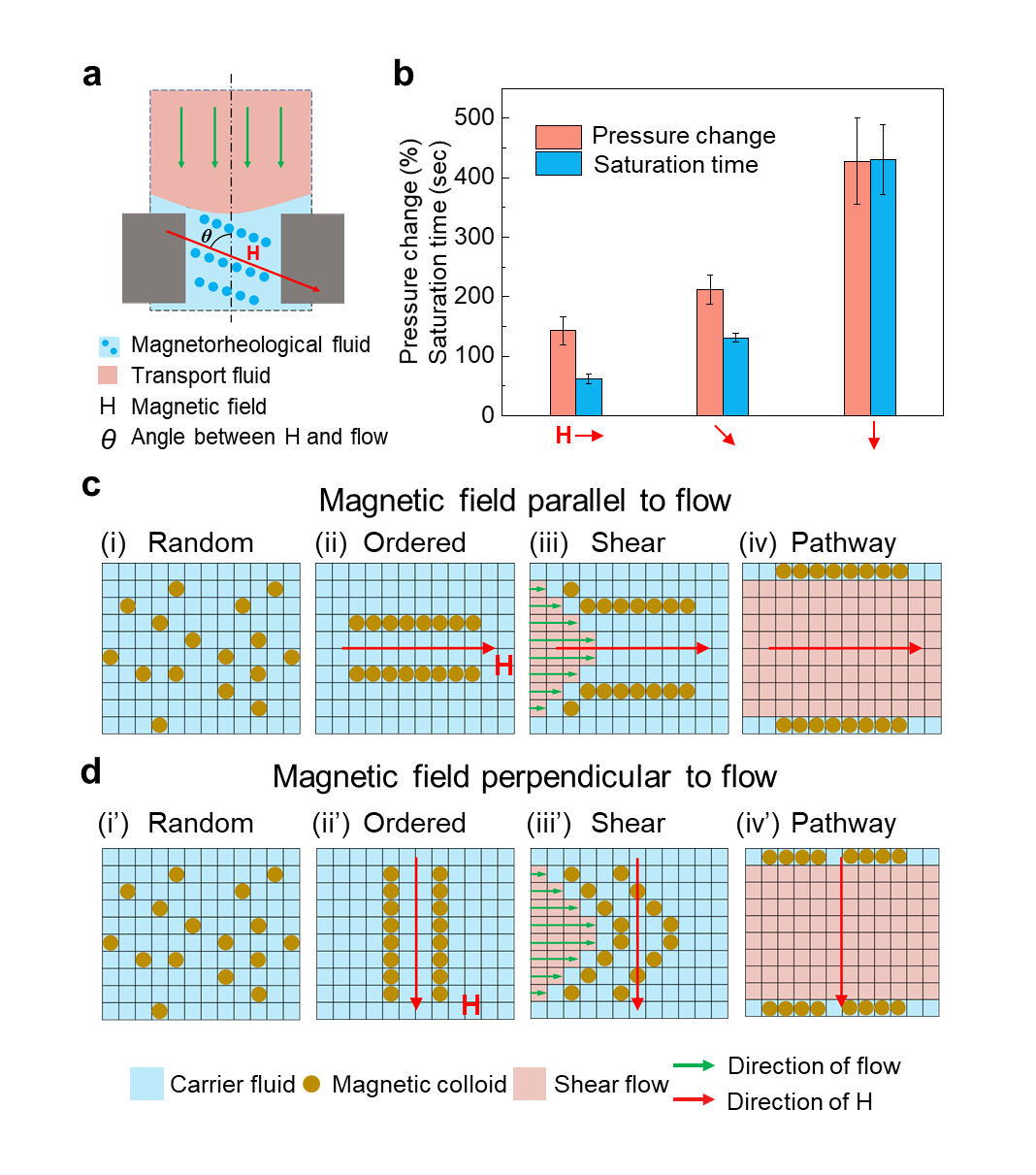


**Supplementary Figure 9. The influence of magnetic direction on the pressure threshold change of gas and saturation time, and the Lattice model analysis.** **a**, Schematic of the correlation between the magnetic direction and flow direction of transport fluid. **b**, Pressure threshold change of gas and saturation time variance as a result of the modulation of magnetic direction. **c**, Based on the lattice model, the four states (random, ordered, shear, and pathway state) of magnetic colloids when the magnetic field is parallel to the transport flow. **d**, Based on the lattice model, the four states (random, ordered, shear, and pathway state) of magnetic colloids when the magnetic field is perpendicular to the transport flow.


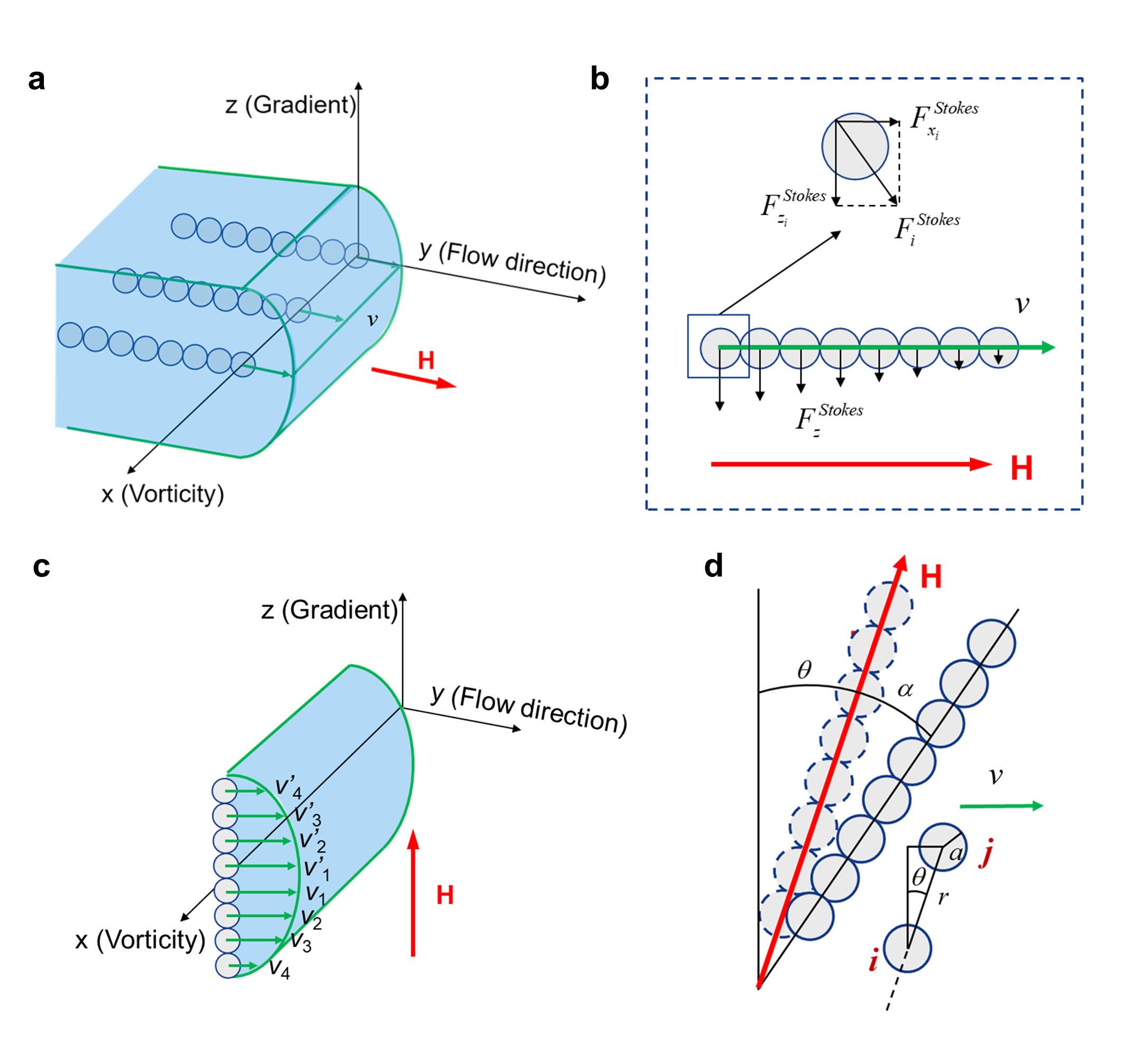


**Supplementary Figure 10. The effect of magnetic direction on the transport flow. a**, Micro-structure of magnetic colloids under the Poiseuille shear flow when the magnetic field is parallel to the flow. **b**, The forces acted on the colloids during the shear flow. **c**, Micro-structure of magnetic colloids under the Poiseuille shear flow when the magnetic field is perpendicular to the flow. **d**, The rod model when calculating the moment of inertia. *θ* represents the angle between the magnetic field and the vertical direction. *α* represents the angle of chain deflection under the joint action of the flow field and magnetic field. a is the radius of the colloid and r is the distance between two colloids *i* and *j*.


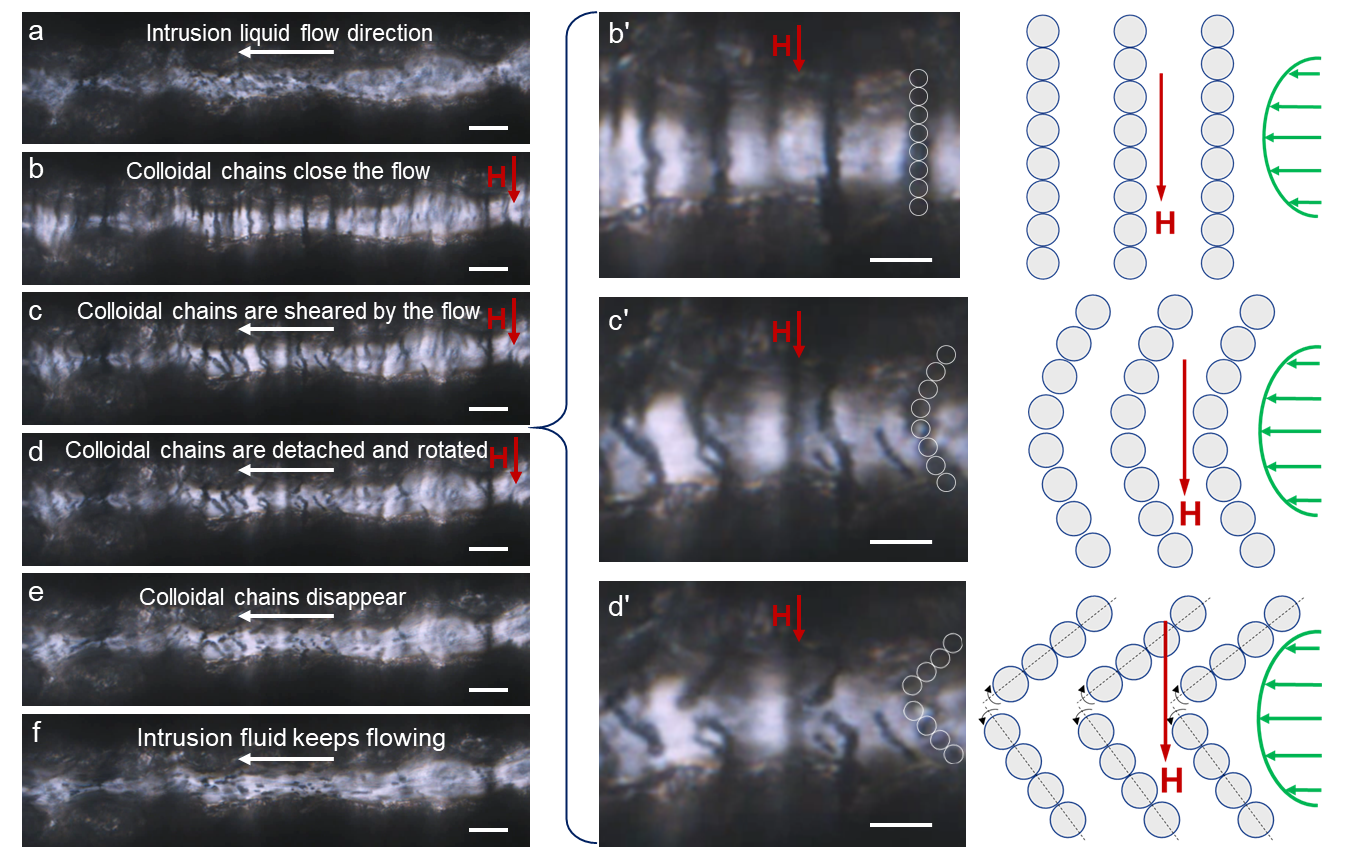


**Supplementary Figure 11. The state of magnetic colloidal chains when encountering an intrusion fluid.** Scale bar is 100 μm in a~f. Scale bar is 50 μm in b’~f’. **a**, Without the magnetic field, the colloidal suspension moves along with the intrusion fluid (Under a given applied pressure, *P*Applied, 0). **b**, Under a magnetic field that is perpendicular to the flow direction, magnetic colloidal chains could close the flow under the same applied pressure *P*Applied, 0. **c**, With increasing the applied pressure (*P*Applied, 1), magnetic colloidal chains are sheared by the flow. **d**, Further increasing the applied pressure (*P*Applied, 2), magnetic colloidal chains are detached and rotated with the flow. **e**, Without the magnetic field, the colloidal chains start to disappear. **f**, Without the magnetic field, the intrusion fluid keeps flowing. b’, c’ and f’ represents the enlarged magnification of the chain structures with their schematics. The circles in b’, c’ and f’ are only for schematics, not for real size.

**
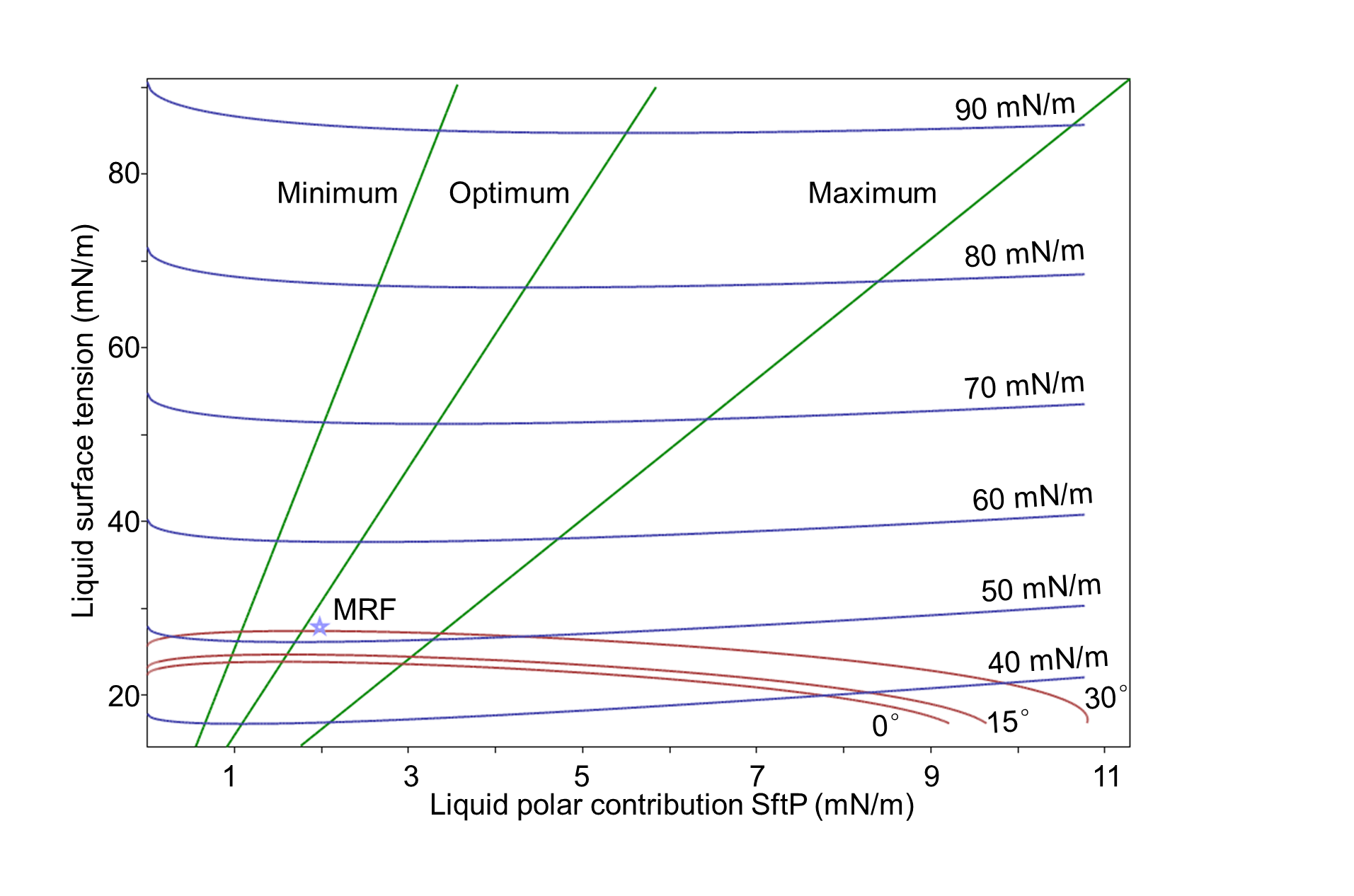
**

**Supplementary Figure 12. Work of adhesion between the magnetorheological fluid and Cu foil.** The purple line provides the ‘isolines’ of work of adhesion of MRF in contact with the Cu foil. The straight green line represents the optimum adhesive force for each level of work. The red lines represent the contact angles, the so-called wetting envelope. MRF falling in the optimum zone indicates that the gating liquid adheres well to the Cu matrix.

**Supplementary Table 1. Stability of the confined magnetic colloid system.** The interfacial energy calculation for three configurations based on the contact angle, surface tension, and interfacial tension. The parameters are measured at room temperature (25°C). The unit of *γ*A, *γ*B, *γ*AB, Δ*E*I, and Δ*E*IIis mN/m. The unit of *θ*Aand *θ*B is degree (°). “Theo.” and “Exp.” stand for theoretical and experimental, respectively.


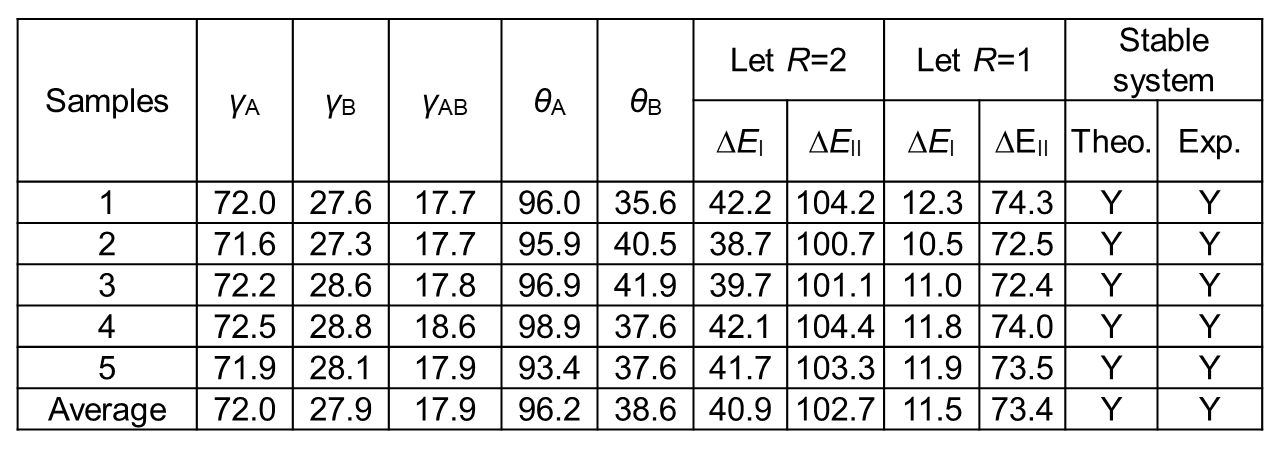


**4. Supplementary movies**

**Movie 1:**

Directional manipulation of confined colloids.

**Movie 2:**

Remote regulation of drug release application.

**Movie 3:**

Remote modulation of microfluidic logic application.

**Movie 4:**

Chemical reaction control application.
